# Supplementary material for: Cytochalasans from the Endophytic Fungus Phomopsis sp. shj2 and Their Antimigratory Activities
Source: J Fungi (Basel). 2022 May 23;8(5):543. doi: 10.3390/jof8050543 (PMC9143583; doi:10.3390/jof8050543)
Supplement: Supplementary file 1 [file jof-08-00543-s001.zip › jof-1730331-supplementary.pdf]

Cytochalasans from the Endophytic Fungus *Phomopsis* sp. shj2 and Their Antimigratory Activities

Bing-Chao Yan [1,2], Wei-Guang Wang [1], Ling-Mei Kong [1], Jian-Wei Tang, Xue Du [1,2], Yan Li [1] and Pema-Tenzin Puno [1,2,*]

[1] State Key Laboratory of Phytochemistry and Plant Resources in West China, Kunming Institute of Botany, Chinese Academy of Sciences, and Yunnan Key Laboratory of Natural Medicinal Chemistry, Kunming 650201, China; yanbingchao@mail.kib.ac.cn (B.-C. Y.); wwg@live.cn (W.-G. W.); konglingmei@mail.kib.ac.cn (L.-M. K.); tangjianwei@mail.kib.ac.cn (J.-W. T.); duxue@mail.kib.ac.cn (X. D.); liyan@mail.kib.ac.cn (Y. L.)

[2] University of Chinese Academy of Sciences, Beijing 100049, China

* Correspondence: punopematenzin@mail.kib.ac.cn (P.-T. P.)

Table of Contents

1. NMR, HRESIMS, UV, ORD, and CD spectra of compound 1 1

2. NMR, HRESIMS, UV, ORD, and CD spectra of compound 2.............7

3. NMR, HRESIMS, UV, ORD, and CD spectra of compound 3..........13

4. NMR, HRESIMS, UV, ORD, and CD spectra of compound 4..........19

5. NMR, HRESIMS, UV, ORD, and CD spectra of compound 5..........25

6. NMR, HRESIMS, UV, ORD, and CD spectra of compound 6..........31

7. NMR, HRESIMS, UV, ORD, and CD spectra of compound 7 37

1. NMR, HRESIMS, UV, ORD, and CD spectra of compound 1

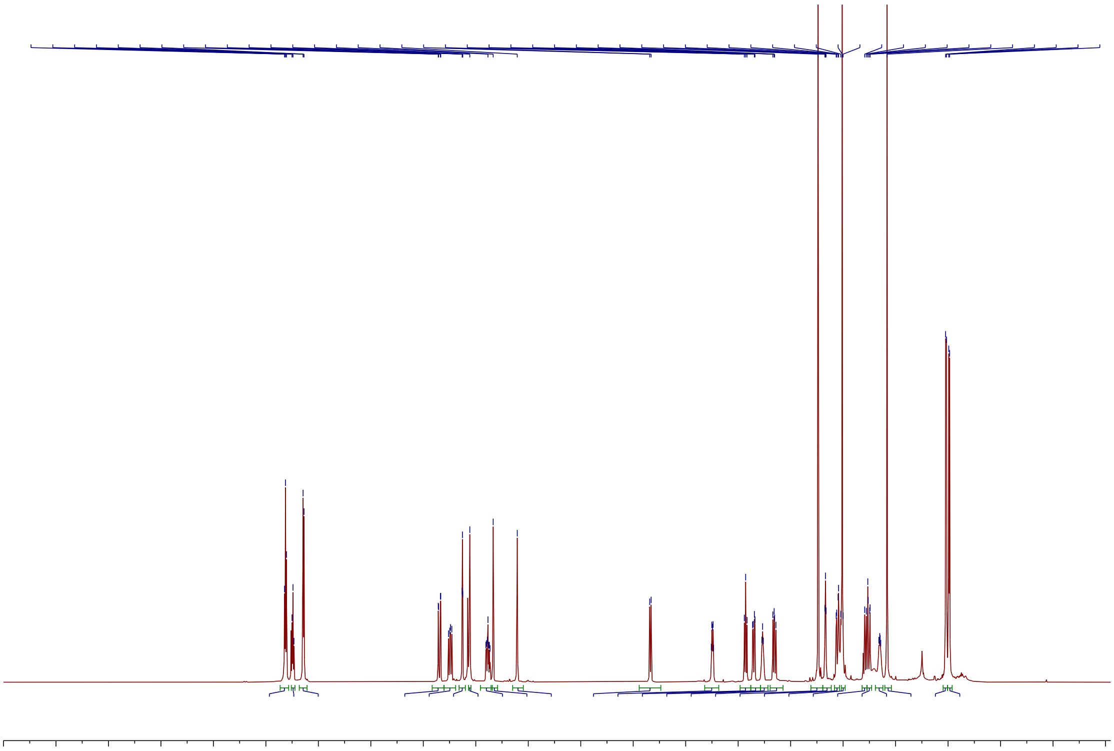

Figure S1. ^{1}H NMR spectrum of **1** (800 MHz, $CDCl_3$).

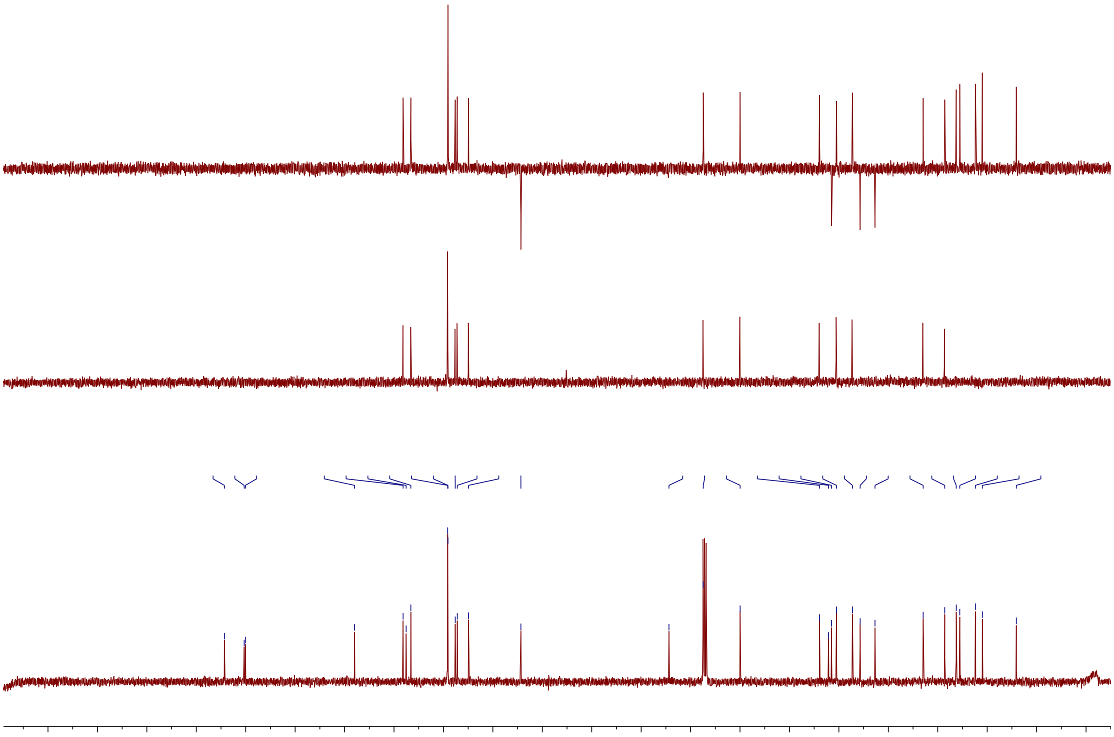

Figure S2. ^{13}C NMR, DEPT-90 and DEPT-135 spectra of **1** (100 MHz, $CDCl_3$).

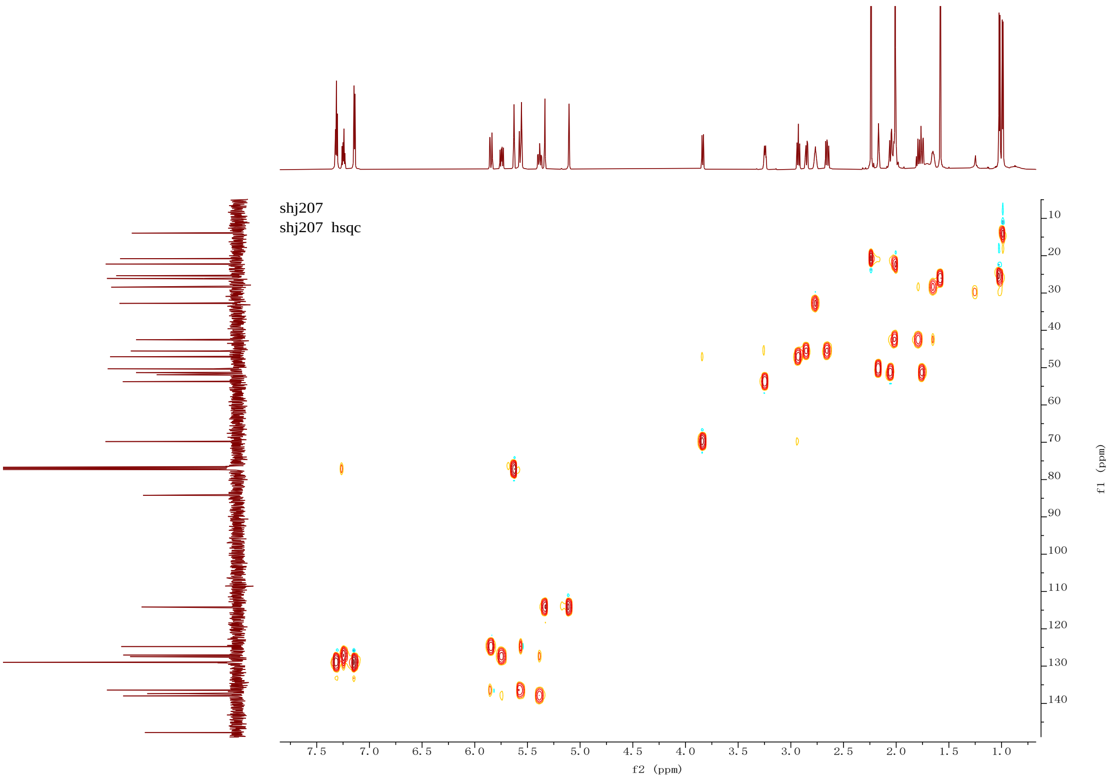

Figure S3. HSQC spectrum of **1** (400 MHz, $CDCl_3$).

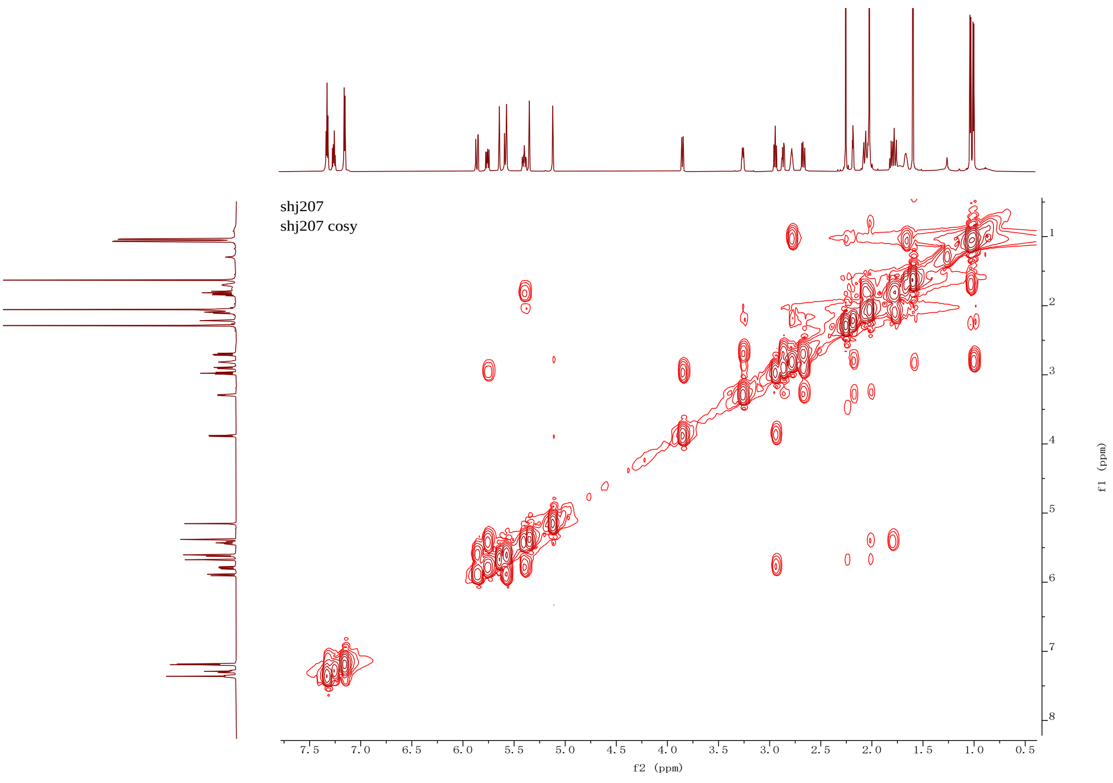

Figure S4. ^{1}H-^{1}H COSY spectrum of **1** (400 MHz, $CDCl_3$).

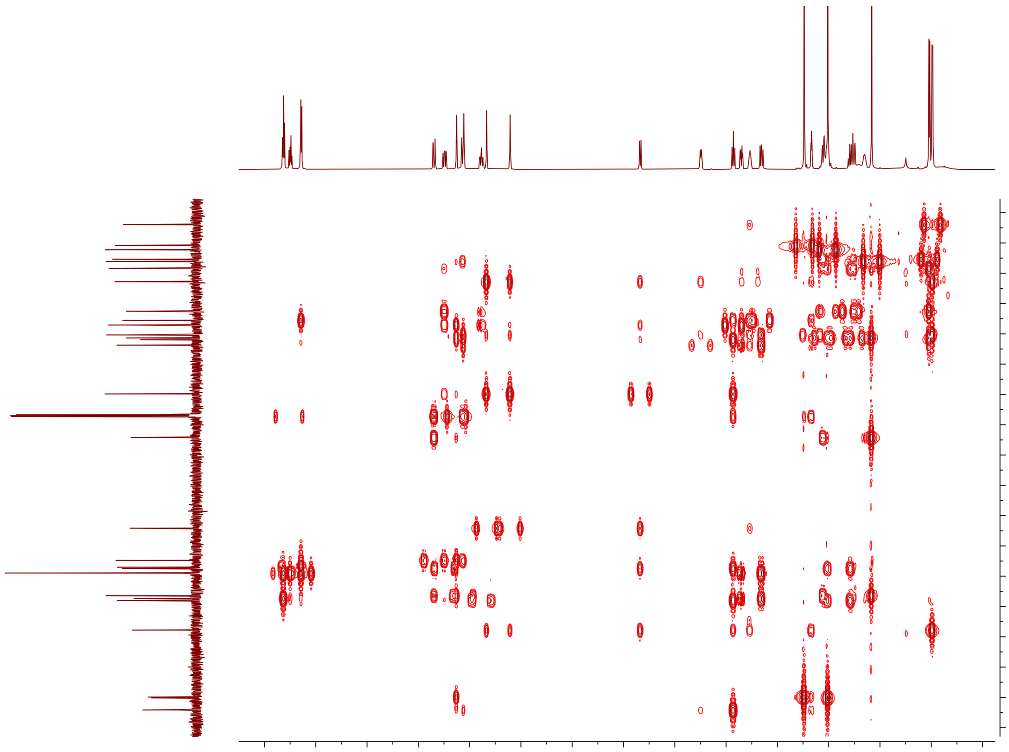

Figure S5. HMBC spectrum of **1** (400 MHz, $CDCl_3$).

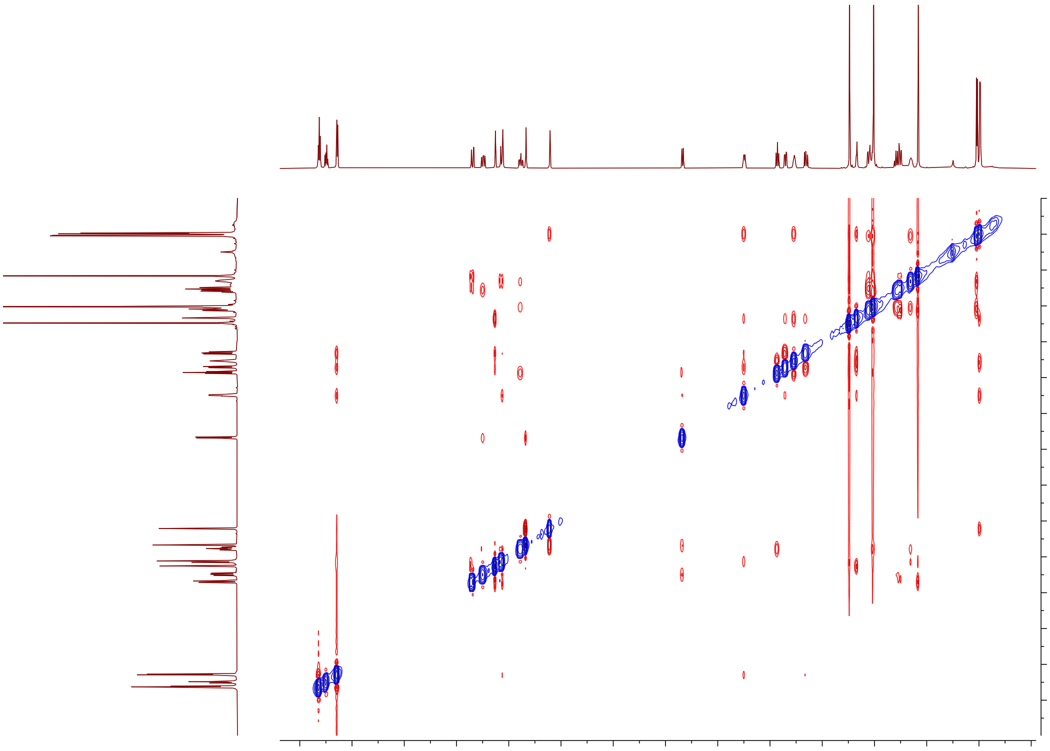

Figure S6. ROESY spectrum of **1** (400 MHz, $CDCl_3$).

Qualitative Analysis Report

**Data Filename**	SHJ207.d	**Sample Name**	SHJ207
**Sample Type**	Sample	**Position**	P1-A1
**Instrument Name**	Instrument 1	**User Name**	
**Acq Method**	SIBU.m	**Acquired Time**	12/23/2014 9:20:46 AM
**IRM Calibration Status**	Success	**DA Method**	Default.m
**Comment**			

**Sample Group**		**Info.**
**Acquisition SW Version**	6200 series TOF/6500 series Q-TOF B.05.01 (B5125.2)	

User Spectra

Fragmentor Voltage	Collision Energy	Ionization Mode
135	0	ESI

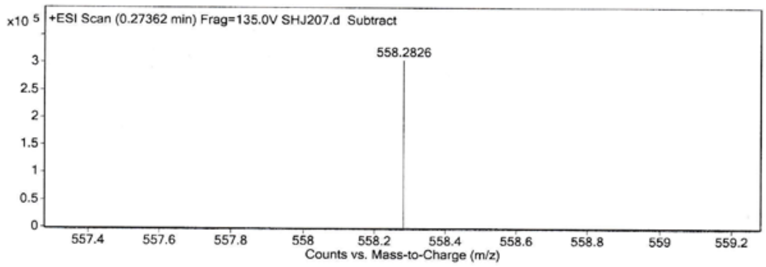

Peak List

m/z	z	Abund	Formula	Ion
476.279	1	101074.23		
477.2823	1	33766.22		
536.3005	1	59321.33		
537.3039	1	20107.72		
558.2826	1	304737.38	C32 H41 N O6	(M+Na)+
559.2854	1	104583.77	C32 H41 N O6	(M+Na)+
560.2875	1	20597.91	C32 H41 N O6	(M+Na)+
574.2633	1	18297.42		
1093.5742	1	41656.8		
1094.5808	1	30091.71		

Formula Calculator Element Limits

Element	Min	Max
C	3	60
H	0	120
O	0	10
N	0	3

Formula Calculator Results

Formula	CalculatedMass	CalculatedMz	Mz	Diff. (mDa)	Diff. (ppm)	DBE
C32 H41 N O6	535.2934	558.2826	558.2826	0.2	0.4	13.0000

--- End Of Report ---

Agilent Technologies Page 1 of 1 Printed at: 10:01 AM on: 12/23/2014

Figure S7. HRESIMS spectrum of **1**.

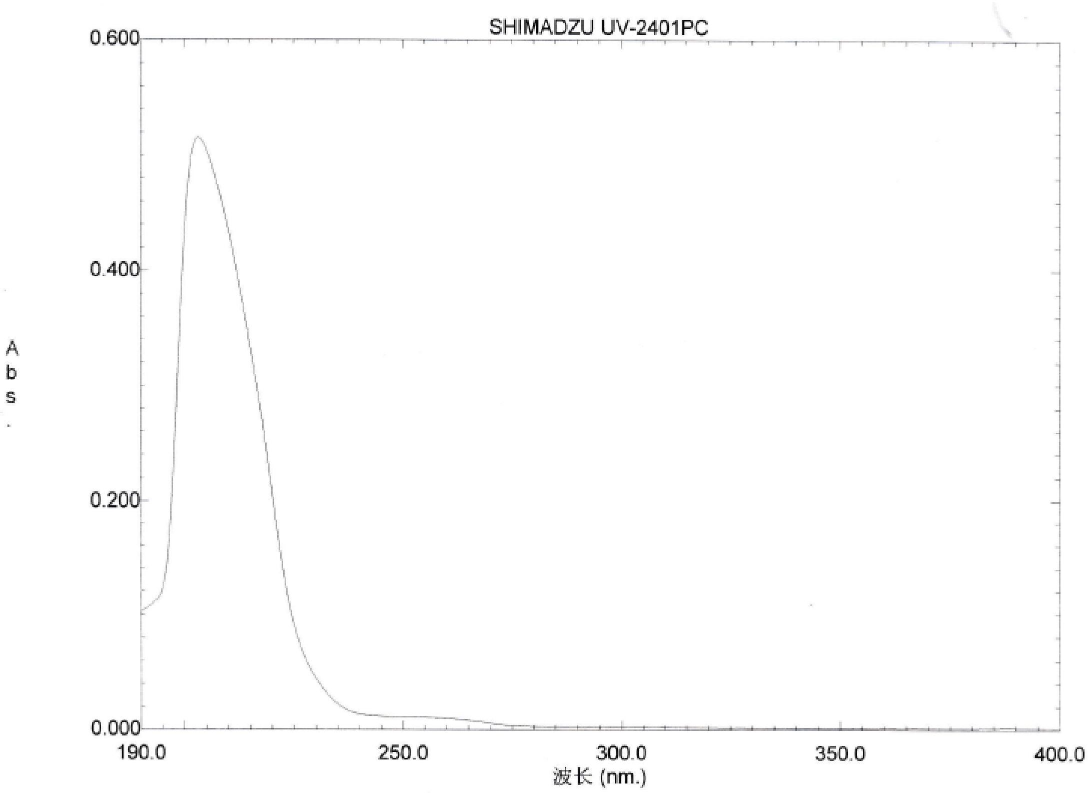

文件名: SHJ207

SHJ207 ———

样品浓度：0.0128毫克/毫升
溶剂：甲醇

创建于: 15:12 15-01-23
数据: 原始

测量模式: Abs.
扫描速度: 中速
狭缝: 5.0
采样间隔: 0.2

否.	波长 (nm.)	Abs.
1	203.20	0.5151

Figure S8. UV spectrum of **1**.

Optical rotation measurement

Model : P-1020 (A060460638)

No.	Sample	Mode	Data	Monitor<br>Blank	Temp.<br>Cell<br>Temp Point	Date<br>Comment<br>Sample Name	Light<br>Filter<br>Operator	Cycle Time<br>Integ Time
No.1	20 (1/3)	Sp.Rot	44.3860	0.0506<br>0.0000	19.6<br>50.00<br>Cell	Thu Jan 22 18:11:51 2015<br>0.00228g/mL MeOH<br>SHJ207	Na<br>589nm	2 sec<br>10 sec
No.2	20 (2/3)	Sp.Rot	45.2630	0.0516<br>0.0000	19.6<br>50.00<br>Cell	Thu Jan 22 18:12:04 2015<br>0.00228g/mL MeOH<br>SHJ207	Na<br>589nm	2 sec<br>10 sec
No.3	20 (3/3)	Sp.Rot	42.8070	0.0488<br>0.0000	19.6<br>50.00<br>Cell	Thu Jan 22 18:12:18 2015<br>0.00228g/mL MeOH<br>SHJ207	Na<br>589nm	2 sec<br>10 sec

+44.150°

Figure S9. ORD spectrum of **1**.

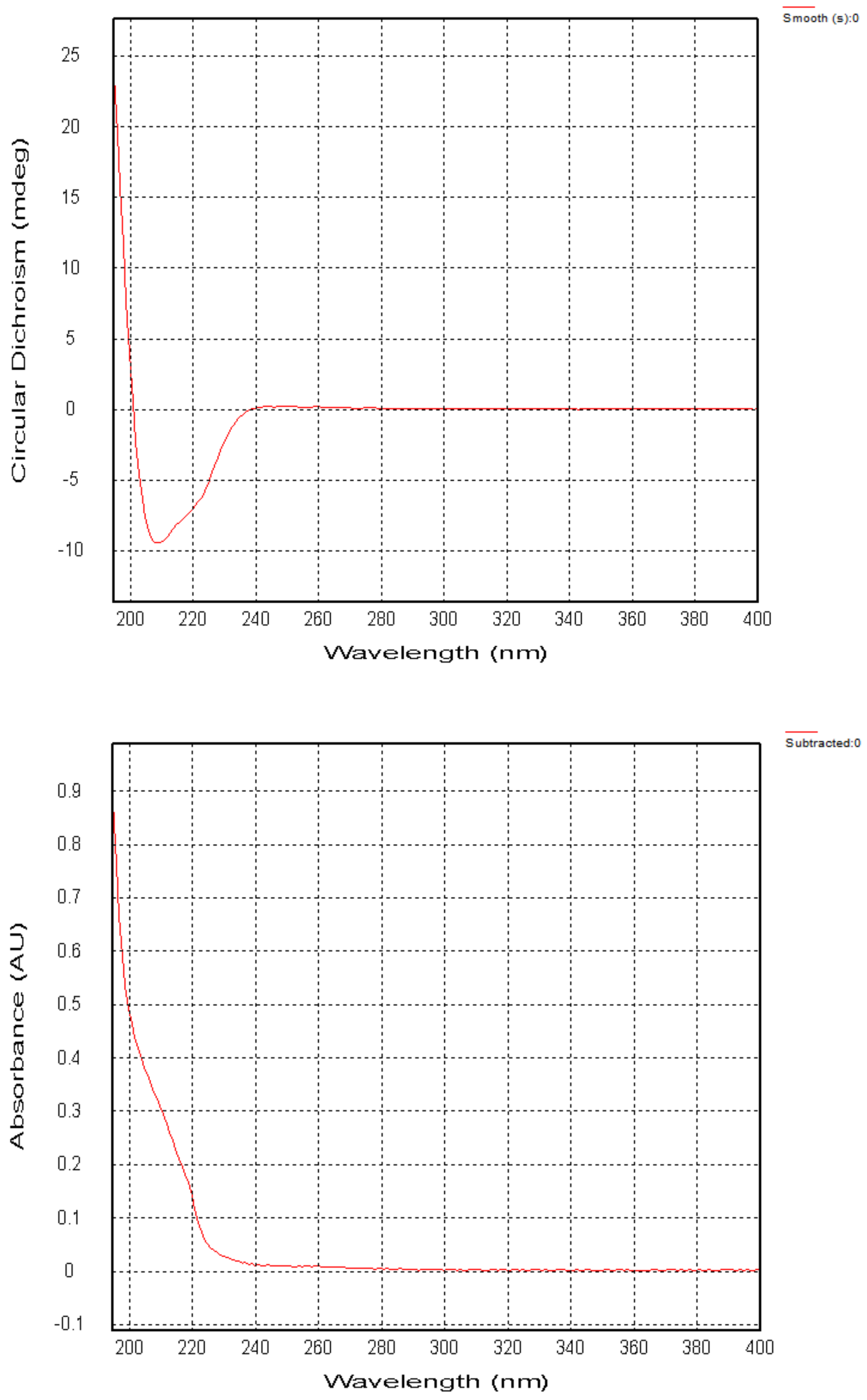

Figure S10. CD spectrum of **1**.

2. NMR, HRESIMS, UV, ORD, and CD spectra of compound 2

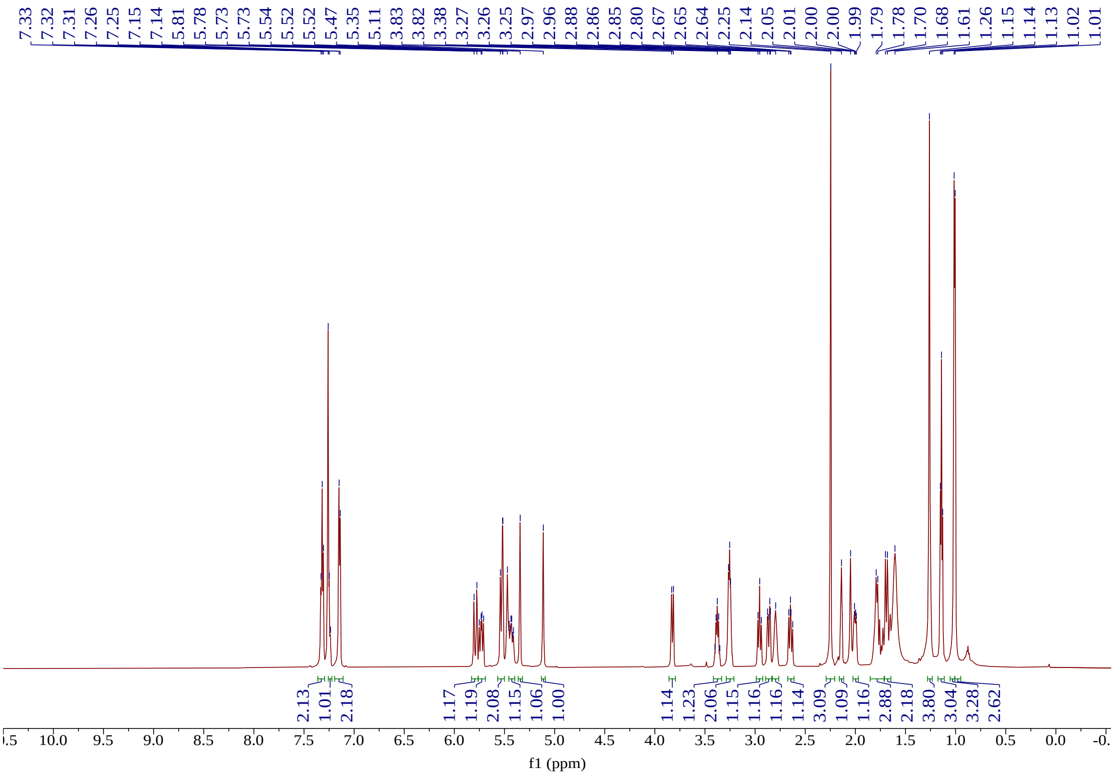

Figure S10. ^{1}H NMR spectrum of **2** (600 MHz, $CDCl_3$).

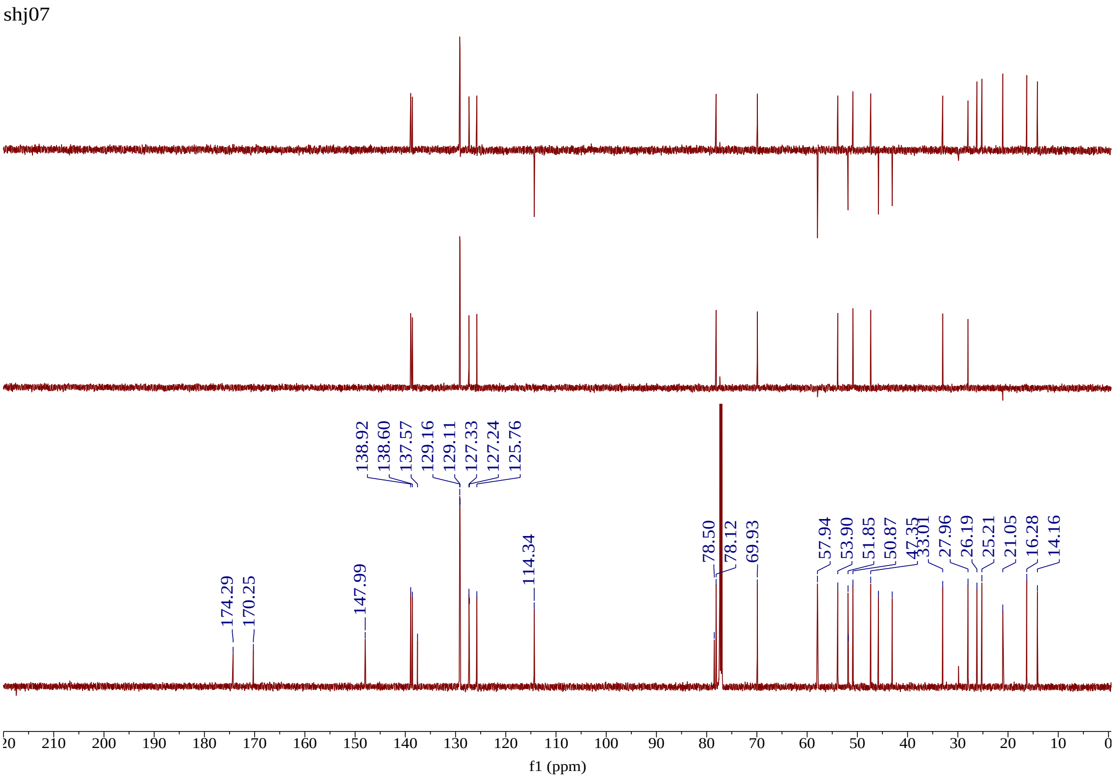

Figure S11. ^{13}C NMR, DEPT-90 and DEPT-135 spectra of **2** (150 MHz, $CDCl_3$).

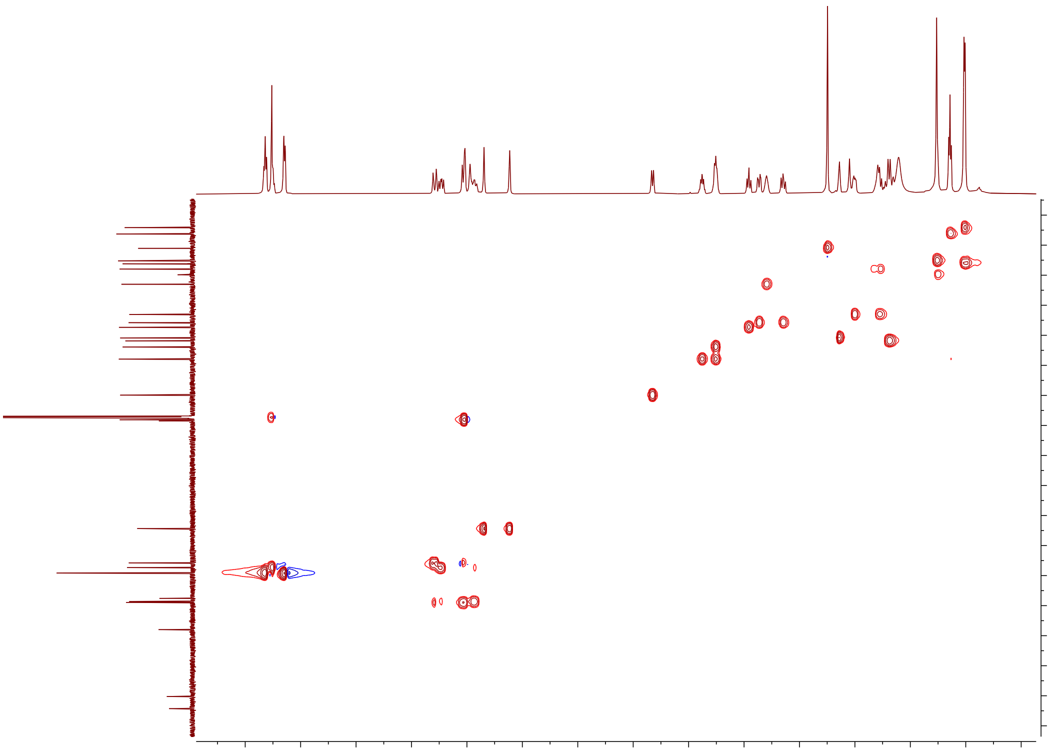

Figure S12. HSQC spectrum of **2** (600 MHz, $CDCl_3$).

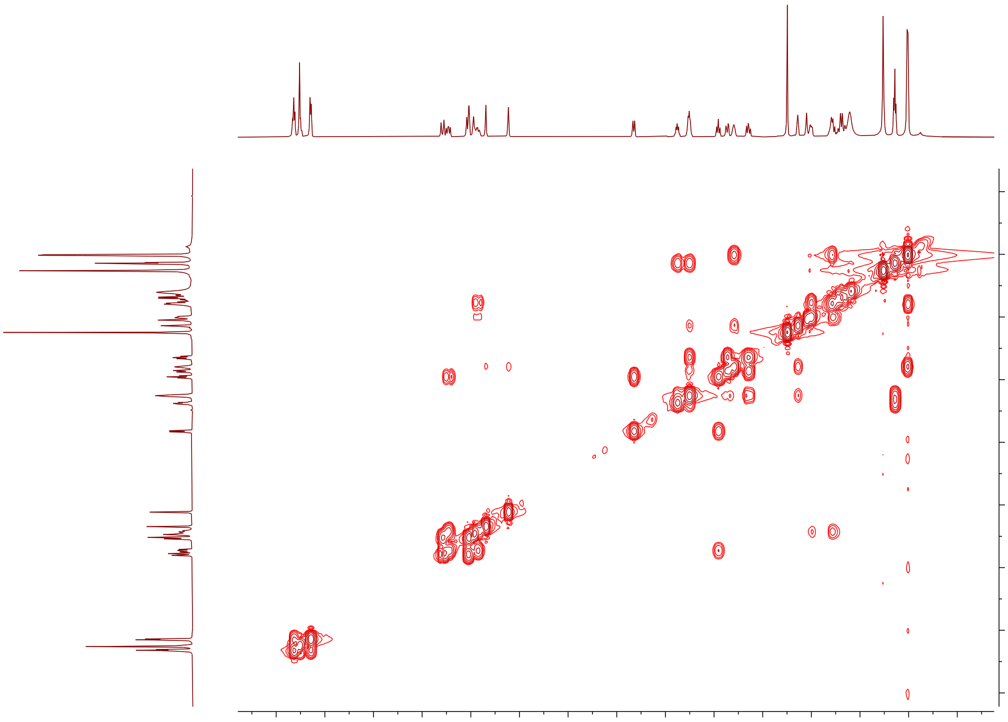

Figure S13. ^{1}H-^{1}H COSY spectrum of **2** (600 MHz, $CDCl_3$).

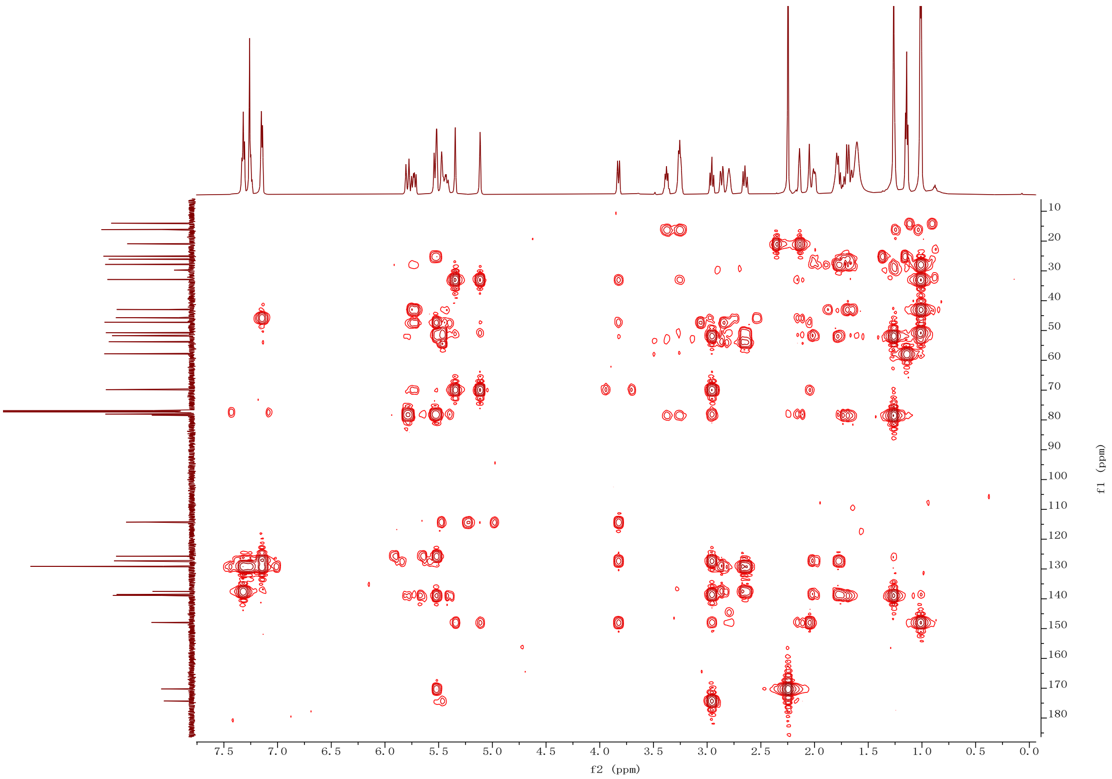

Figure S14. HMBC spectrum of **2** (600 MHz, $CDCl_3$).

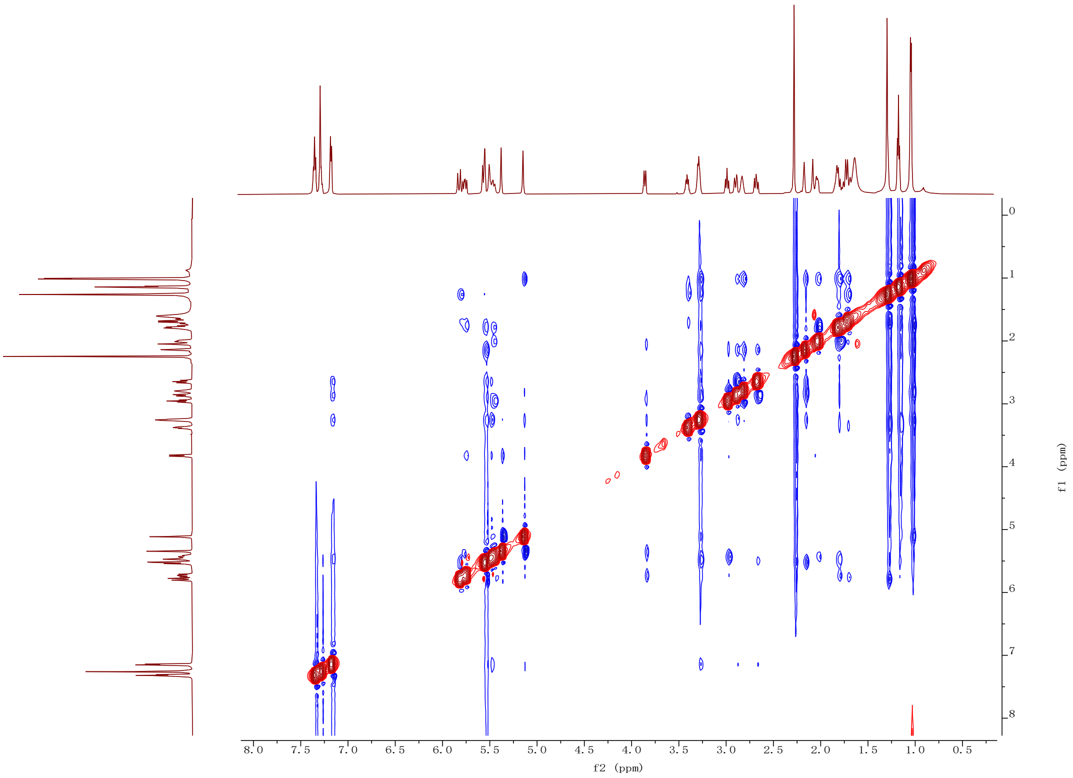

Figure S15. ROESY spectrum of **2** (600 MHz, $CDCl_3$).

Qualitative Analysis Report

**Data Filename**	141104ESIA2.d	**Sample Name**	SHJ07
**Sample Type**	Sample	**Position**	
**Instrument Name**	Agilent G6230 TOF MS	**User Name**	KIB
**Acq Method**	ESI.m	**Acquired Time**	11/4/2014 10:08:07 AM
**IRM Calibration Status**	Success	**DA Method**	demo.m
**Comment**			

Sample Group **Info.**

Acquisition SW 6200 series TOF/6500 series
Version Q-TOF B.05.01 (B5125.2)

User Spectra

Fragmentor Voltage	Collision Energy	Ionization Mode
200	0	ESI

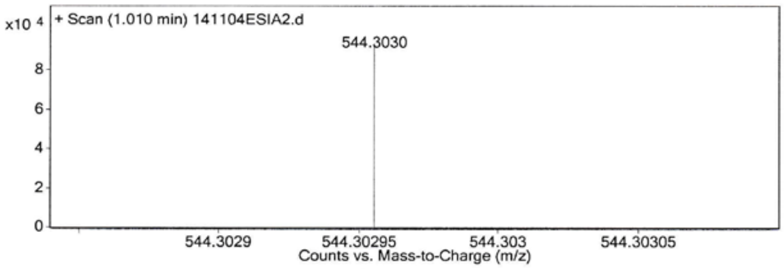

Peak List

m/z	z	Abund	Formula	Ion
544.303	1	93731.61	C32 H43 N Na O5	M+

Formula Calculator Element Limits

Element	Min	Max
C	0	200
H	0	400
O	1	8
Na	1	1
N	1	1

Formula Calculator Results

Formula	CalculatedMass	Mz	Diff.(mDa)	Diff. (ppm)	DBE
C32 H43 N Na O5	544.3039	544.3030	0.9	1.6	11.5

--- End Of Report ---

Figure S16. HRESIMS spectrum of **2**.

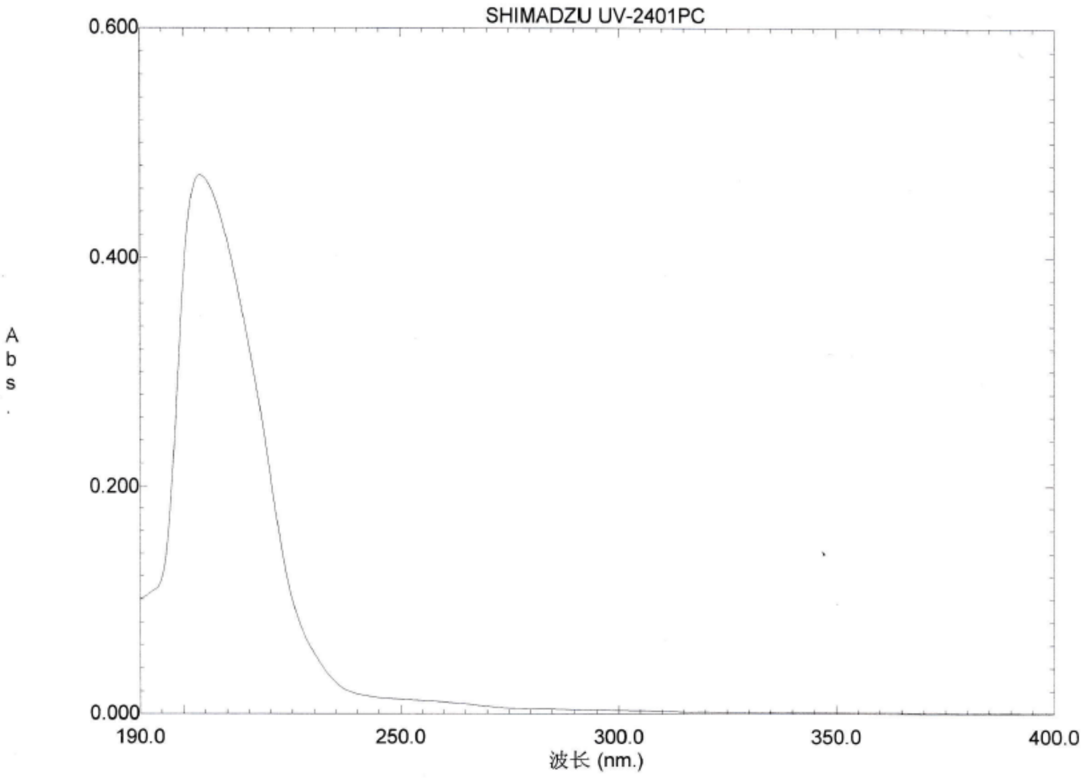

文件名: SHJ07

SHJ07 ———

样品浓度：0.0111毫克/毫升
溶剂：甲醇

创建于: 15:53 15-01-21
数据: 原始

测量模式: Abs.
扫描速度: 中速
狭缝: 5.0
采样间隔: 0.2

否.	波长 (nm.)	Abs.
1	204.00	0.4717

Figure S17. UV spectrum of **2**.

Optical rotation measurement

Model : P-1020 (A060460638)

No.	Sample	Mode	Data	Monitor<br>Blank	Temp.<br>Cell<br>Temp Point	Date<br>Comment<br>Sample Name	Light<br>Filter<br>Operator	Cycle Time<br>Integ Time
No.1	17 (1/3)	Sp.Rot	38.5710	0.0297<br>0.0000	18.1<br>50.00<br>Cell	Tue Jan 20 20:57:47 2015<br>0.00154g/mL MeOH<br>SHJ07	Na<br>589nm	2 sec<br>10 sec
No.2	17 (2/3)	Sp.Rot	39.3510	0.0303<br>0.0000	18.1<br>50.00<br>Cell	Tue Jan 20 20:58:00 2015<br>0.00154g/mL MeOH<br>SHJ07	Na<br>589nm	2 sec<br>10 sec
No.3	17 (3/3)	Sp.Rot	38.9610	0.0300<br>0.0000	18.1<br>50.00<br>Cell	Tue Jan 20 20:58:13 2015<br>0.00154g/mL MeOH<br>SHJ07	Na<br>589nm	2 sec<br>10 sec

+38.9610°

Figure S18. ORD spectrum of **2**.

SHJ07

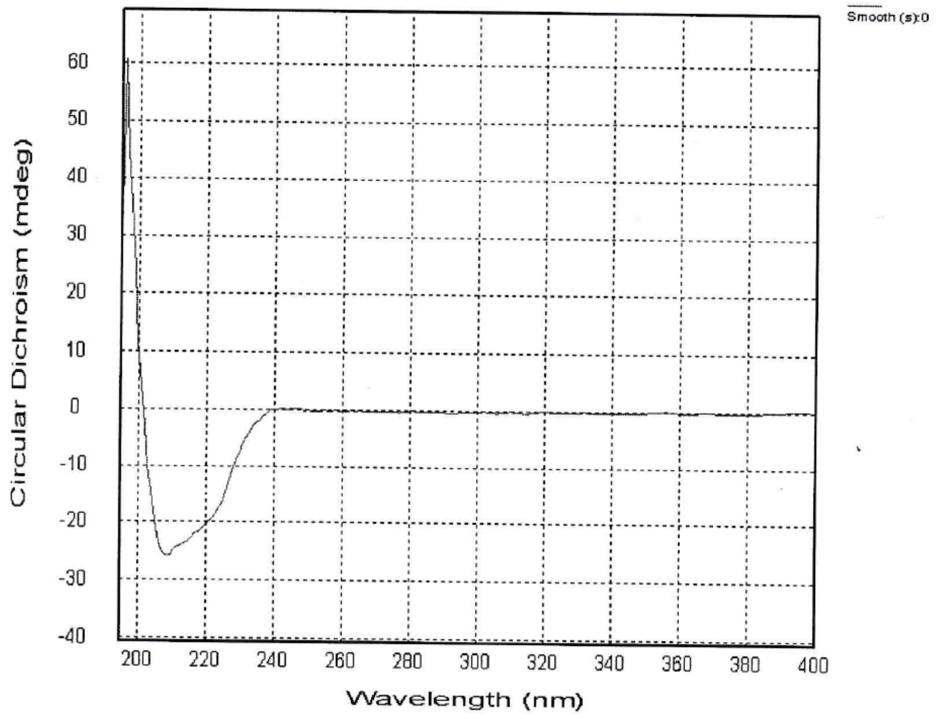

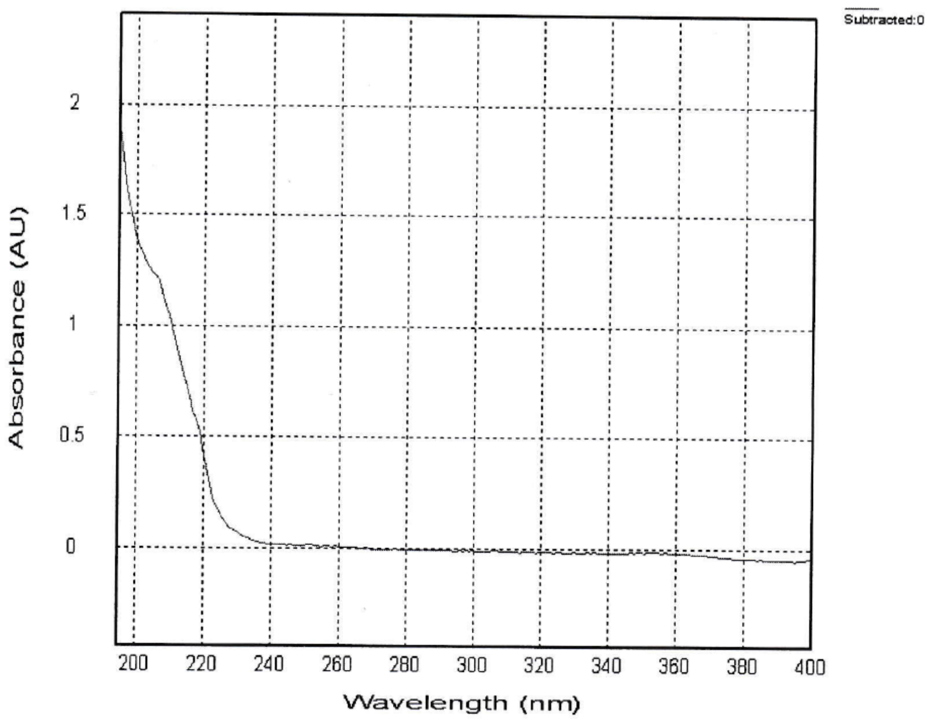

Figure S19. CD spectrum of **2**.

3. NMR, HRESIMS, UV, ORD, and CD spectra of compound 3

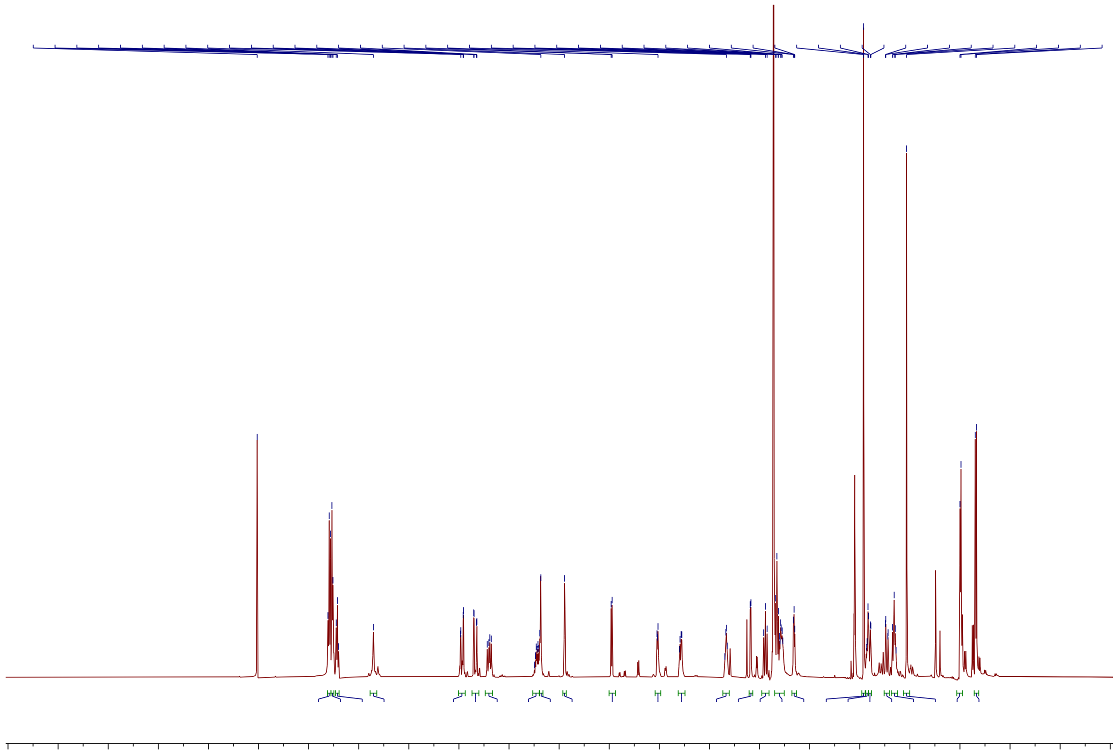

Figure S20. ^{1}H NMR spectrum of **3** (600 MHz, acetone-d_6).

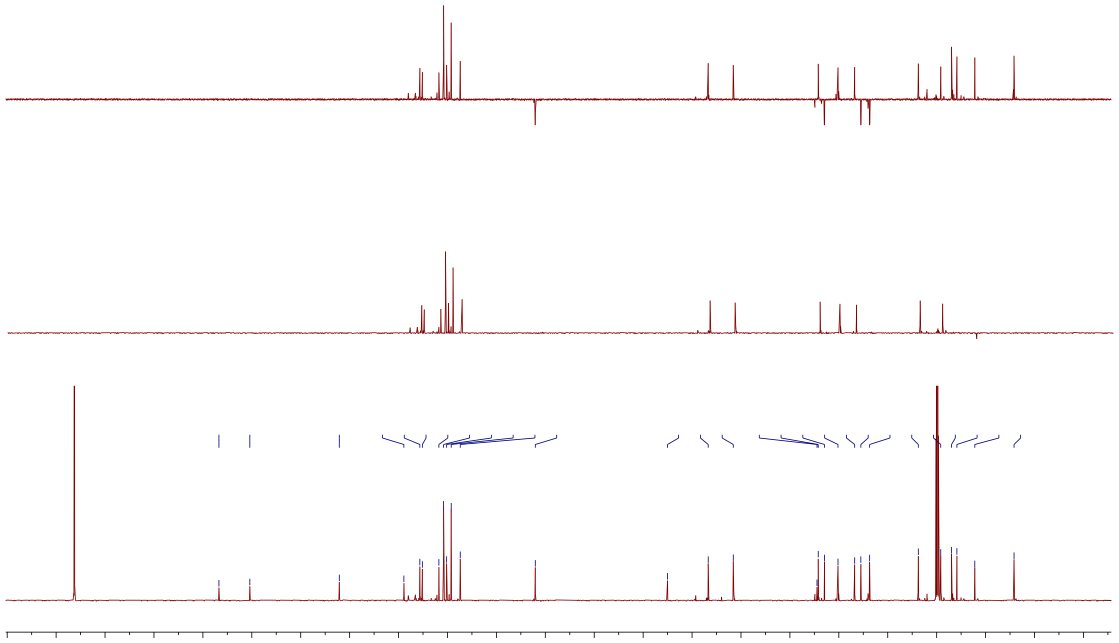

Figure S21. ^{13}C NMR, DEPT-90 and DEPT-135 spectra of **3** (150 MHz, acetone-d_6).

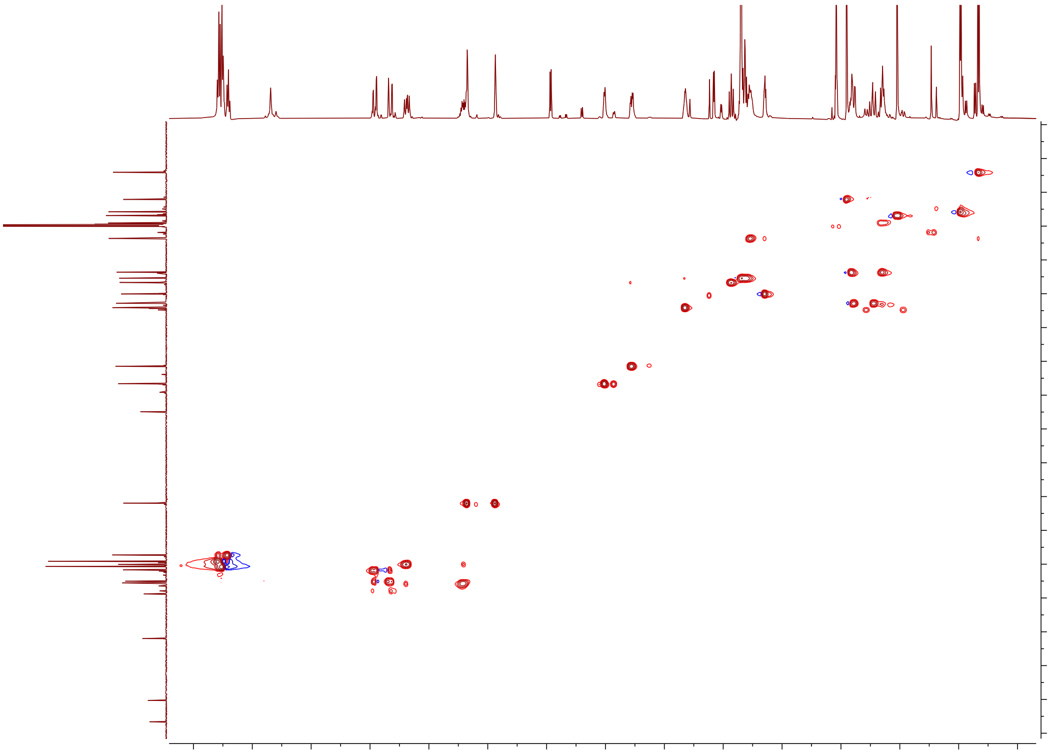

Figure S22. HSQC spectrum of **3** (600 MHz, acetone-d_6).

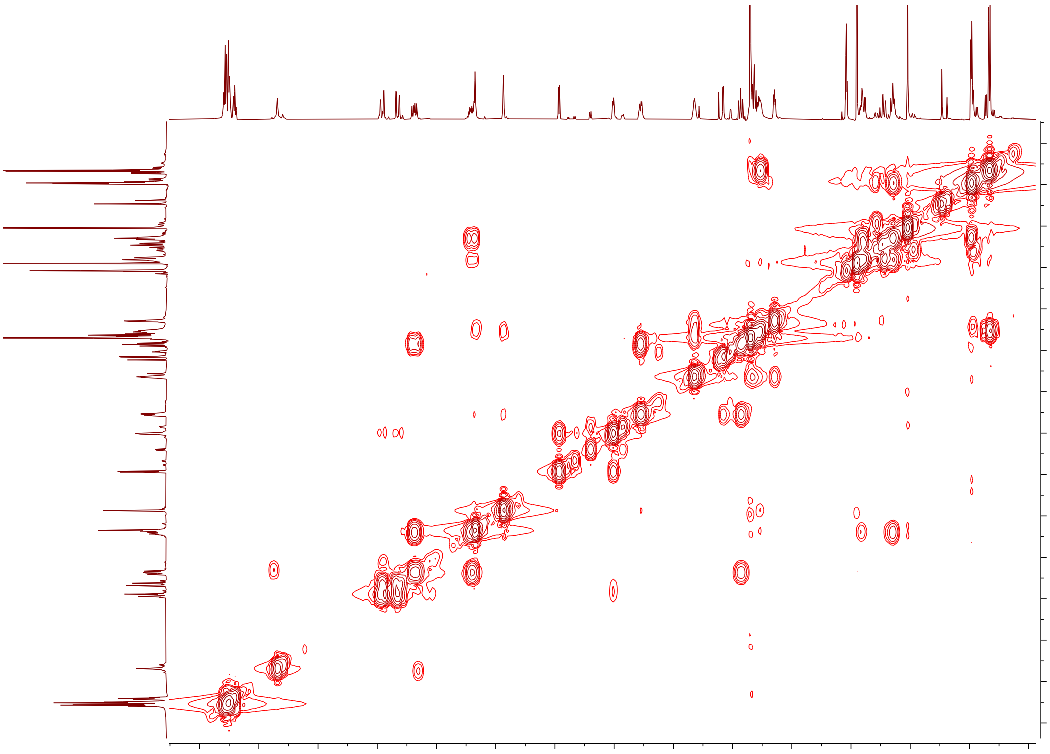

Figure S23. ^{1}H-^{1}H COSY spectrum of **3** (600 MHz, acetone-d_6).

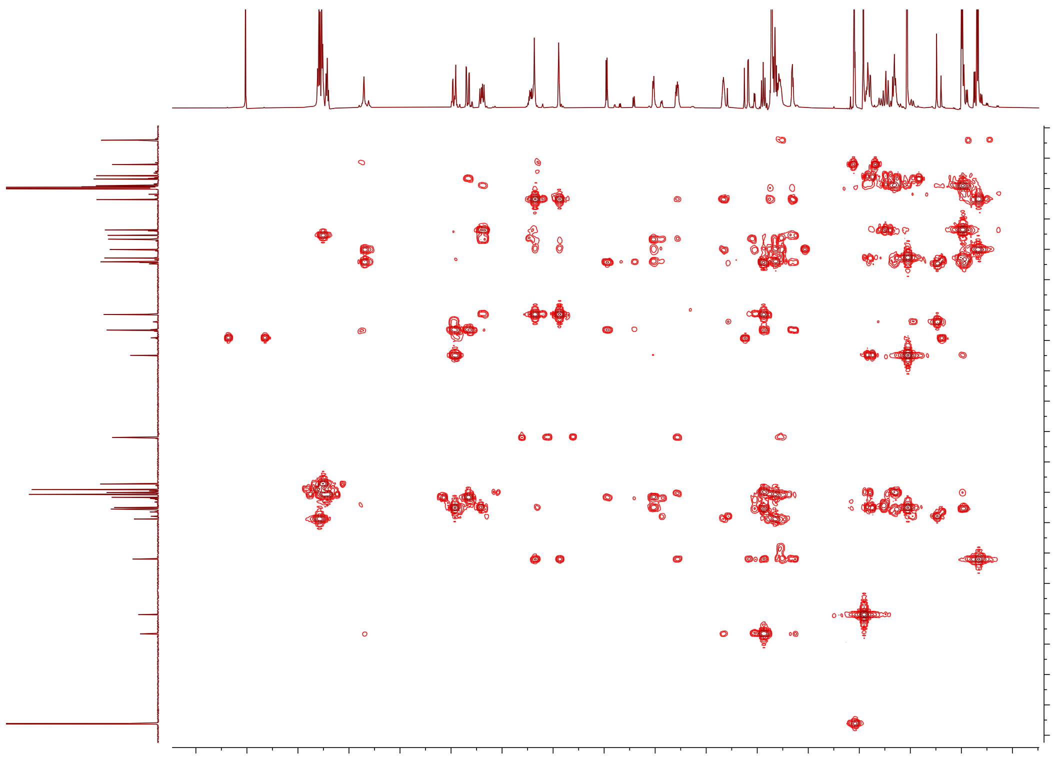

Figure S24. HMBC spectrum of **3** (600 MHz, acetone-d_6).

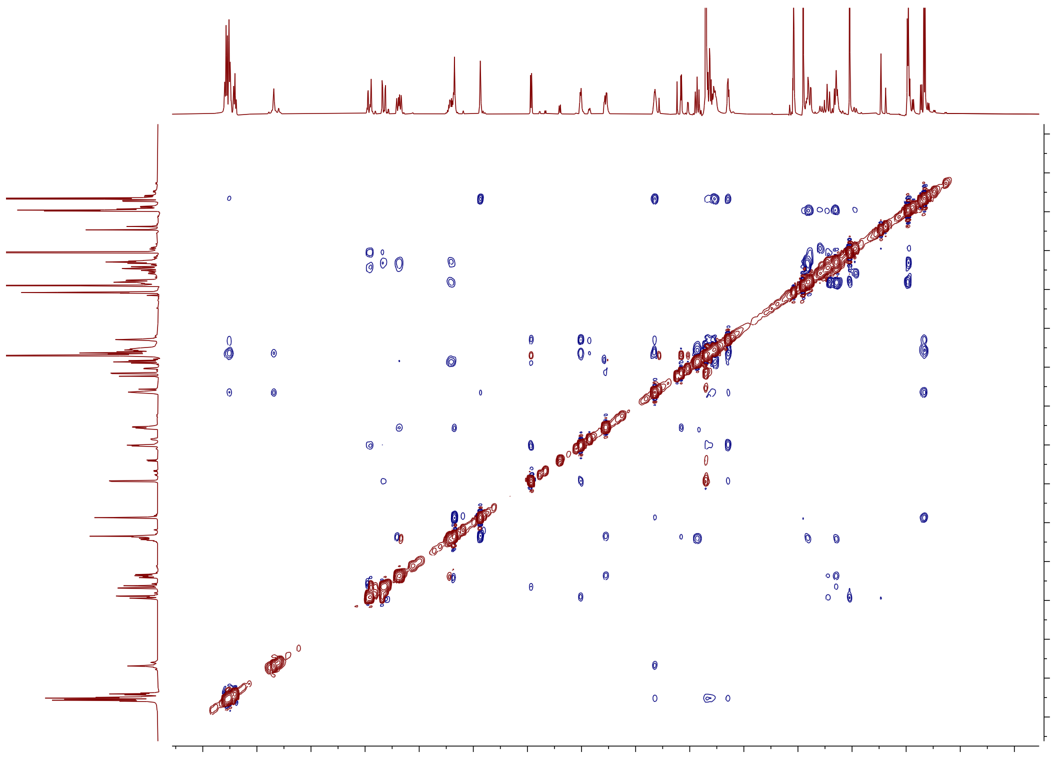

Figure S25. ROESY spectrum of **3** (600 MHz, acetone-d_6).

Qualitative Analysis Report

**Data Filename**	SHJ2061.d	**Sample Name**	SHJ2061
**Sample Type**	Sample	**Position**	P1-B1
**Instrument Name**	Instrument 1	**User Name**	
**Acq Method**	SIBU.m	**Acquired Time**	11/5/2014 10:04:48 AM
**IRM Calibration Status**	Success	**DA Method**	Default.m
**Comment**			

**Sample Group**		**Info.**
**Acquisition SW Version**	6200 series TOF/6500 series Q-TOF B.05.01 (B5125.2)	

User Spectra

Fragmentor Voltage	Collision Energy	Ionization Mode
135	0	ESI

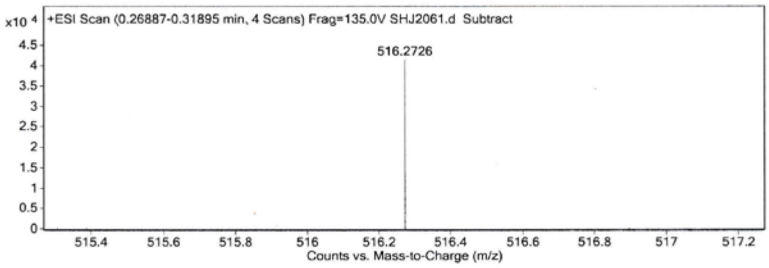

Peak List

m/z	z	Abund	Formula	Ion
301.1415	1	9981.23		
315.1566	1	6578.91		
416.2595	1	6534.67		
434.269	1	14929.77		
435.2731	1	5252.26		
494.2906	1	24097.41		
495.2942	1	8625.36		
516.2726	1	41536.34	C30 H39 N O5	(M+Na)+
517.2759	1	12547.21	C30 H39 N O5	(M+Na)+
652.2481	1	5529.75		

Formula Calculator Element Limits

Element	Min	Max
C	3	60
H	0	120
O	0	30
N	0	5

Formula Calculator Results

Formula	CalculatedMass	CalculatedMz	Mz	Diff. (mDa)	Diff. (ppm)	DBE
C30 H39 N O5	493.2828	516.2720	516.2726	-0.6	-1.2	12.0000

--- End Of Report ---

Agilent Technologies Page 1 of 1 Printed at: 10:10 AM on: 11/5/2014

Figure S26. HRESIMS spectrum of **3**.

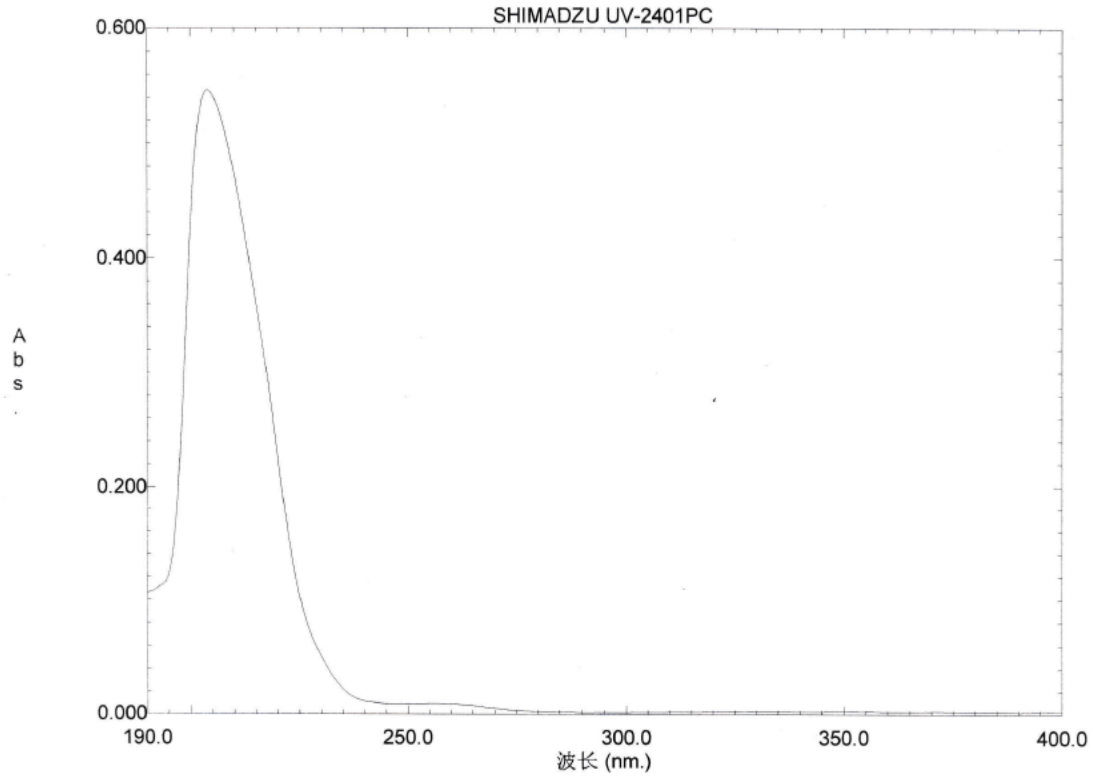

文件名:　15012103
样品名称：SHJ2061

创建于:　16:39　15-01-21
数据:　原始

测量模式:　Abs.
扫描速度:　中速
狭缝:　5.0
采样间隔:　0.2

否.	波长 (nm.)	Abs.
1	204.00	0.5467

15012103———

样品浓度：0.0108毫克/毫升
溶剂：甲醇

Figure S27. UV spectrum of **3**.

Optical rotation measurement

Model : P-1020 (A060460638)

No.	Sample	Mode	Data	Monitor<br>Blank	Temp.<br>Cell<br>Temp Point	Date<br>Comment<br>Sample Name	Light<br>Filter<br>Operator	Cycle Time<br>Integ Time
No.1	8 (1/3)	Sp.Rot	22.4690	0.0273<br>0.0000	21.5<br>50.00<br>Cell	Wed Oct 29 20:34:44 2014<br>0.00243g/mL MeOH<br>SHJ2061	Na<br>589nm	2 sec<br>10 sec
No.2	8 (2/3)	Sp.Rot	20.9880	0.0255<br>0.0000	21.5<br>50.00<br>Cell	Wed Oct 29 20:34:57 2014<br>0.00243g/mL MeOH<br>SHJ2061	Na<br>589nm	2 sec<br>10 sec +22.0027°
No.3	8 (3/3)	Sp.Rot	22.5510	0.0274<br>0.0000	21.6<br>50.00<br>Cell	Wed Oct 29 20:35:10 2014<br>0.00243g/mL MeOH<br>SHJ2061	Na<br>589nm	2 sec<br>10 sec

Figure S28. ORD spectrum of **3**.

SHJ2061

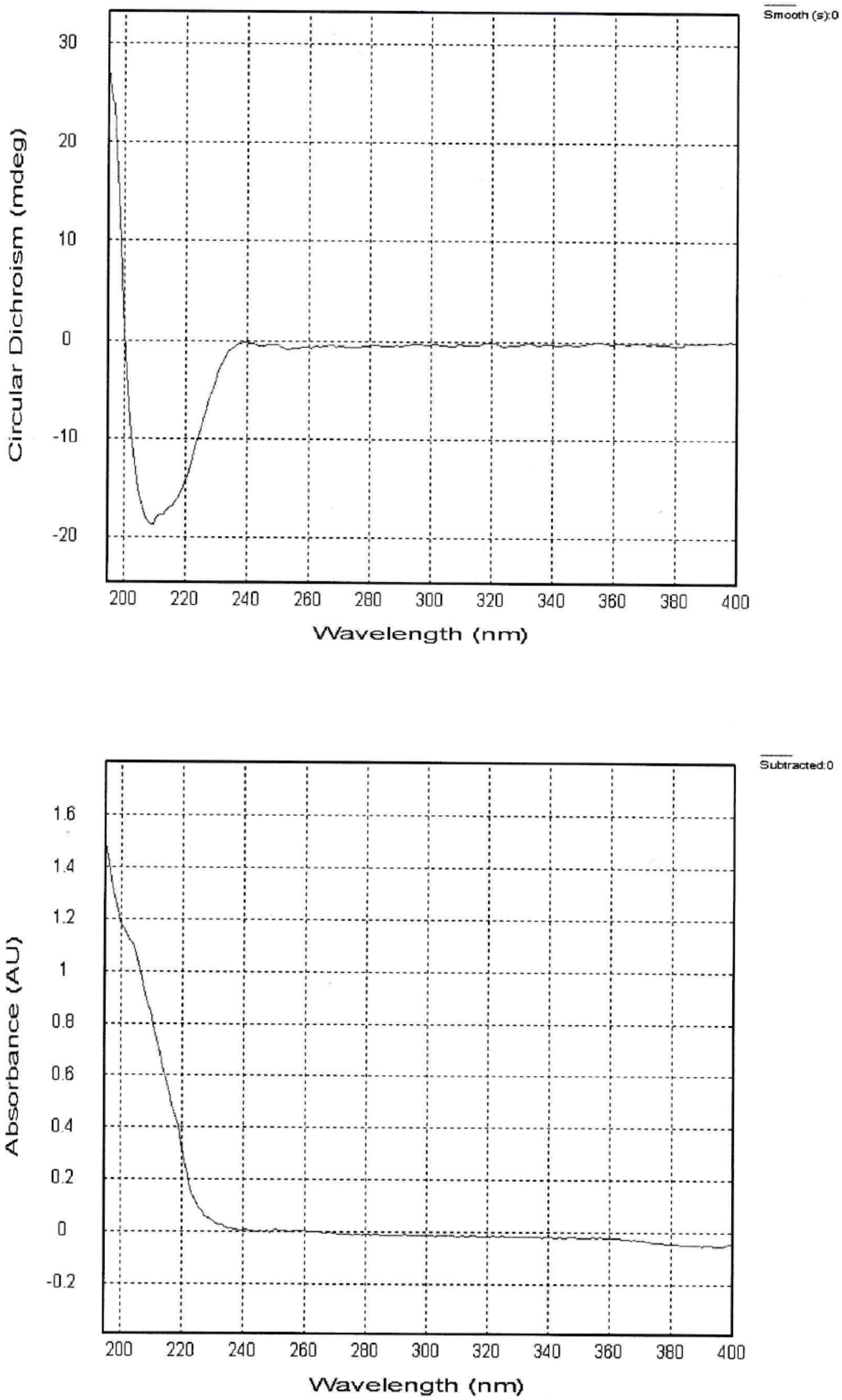

Figure S29. CD spectrum of **3**.

4. NMR, HRESIMS, UV, ORD, and CD spectra of compound 4

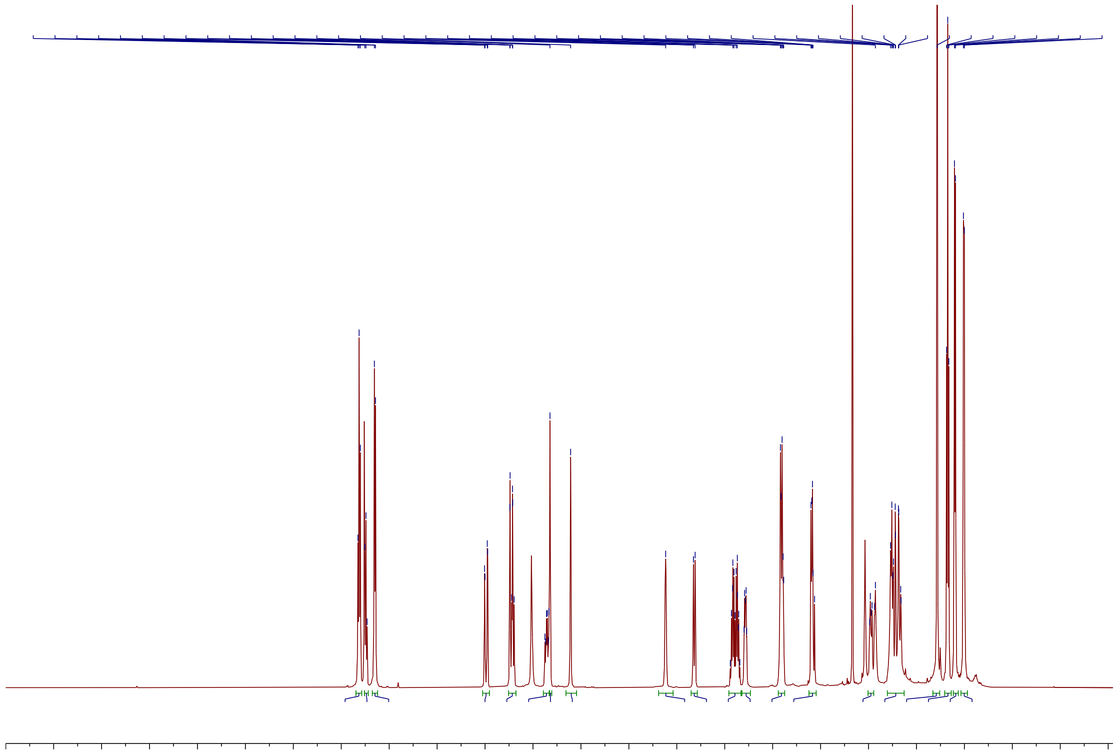

Figure S30. ^{1}H NMR spectrum of **4** (600 MHz, $CDCl_3$).

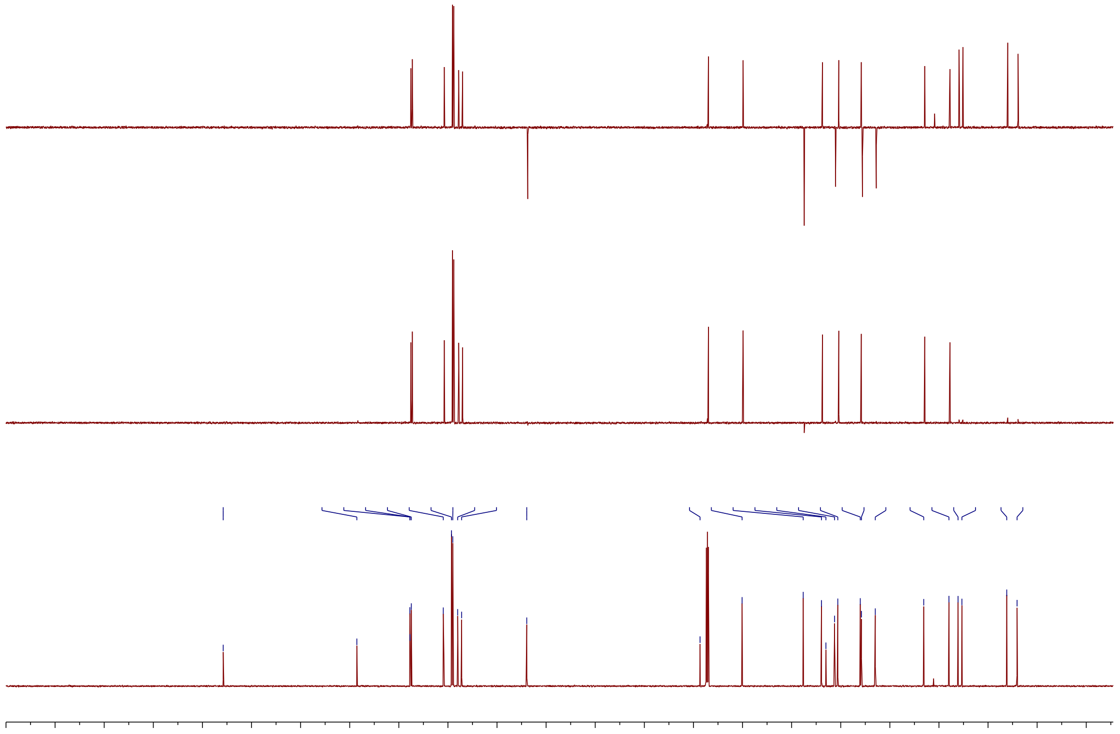

Figure S31. ^{13}C NMR, DEPT-90 and DEPT-135 spectra of **4** (150 MHz, $CDCl_3$).

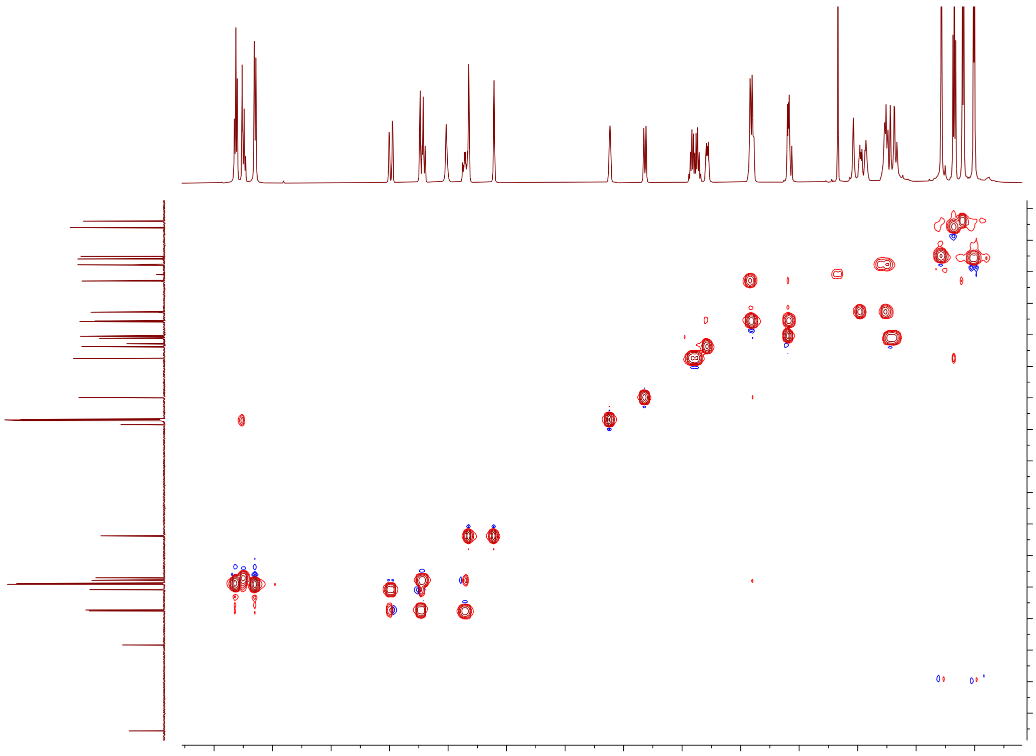

Figure S32. HSQC spectrum of **4** (600 MHz, $CDCl_3$).

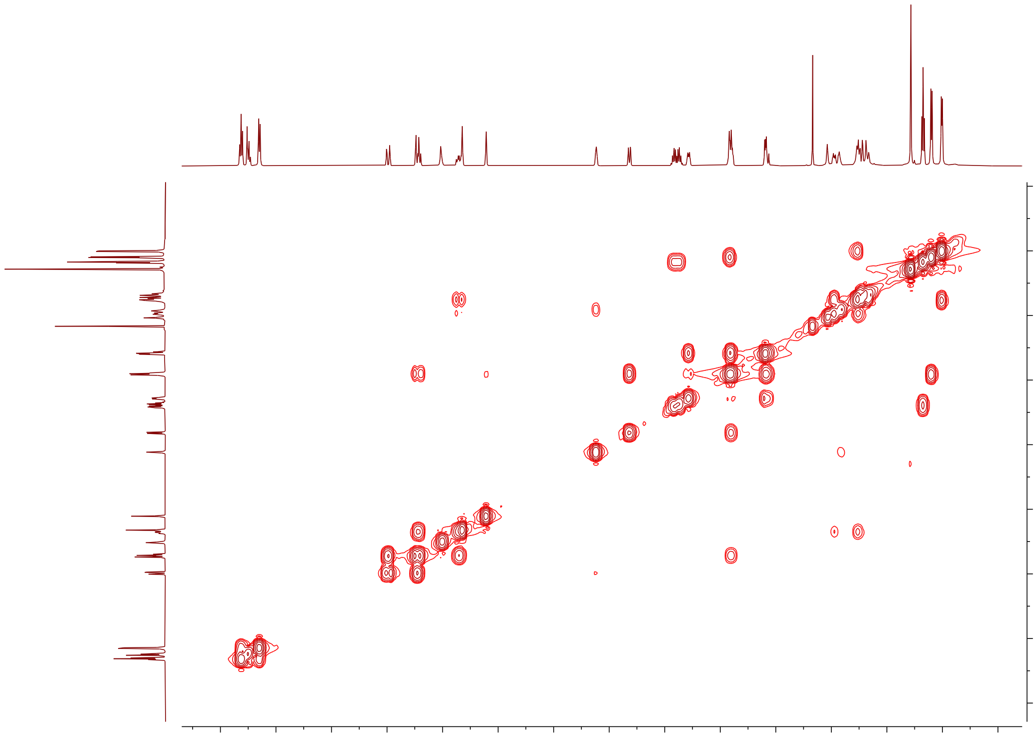

Figure S33. ^{1}H-^{1}H COSY spectrum of **4** (600 MHz, $CDCl_3$).

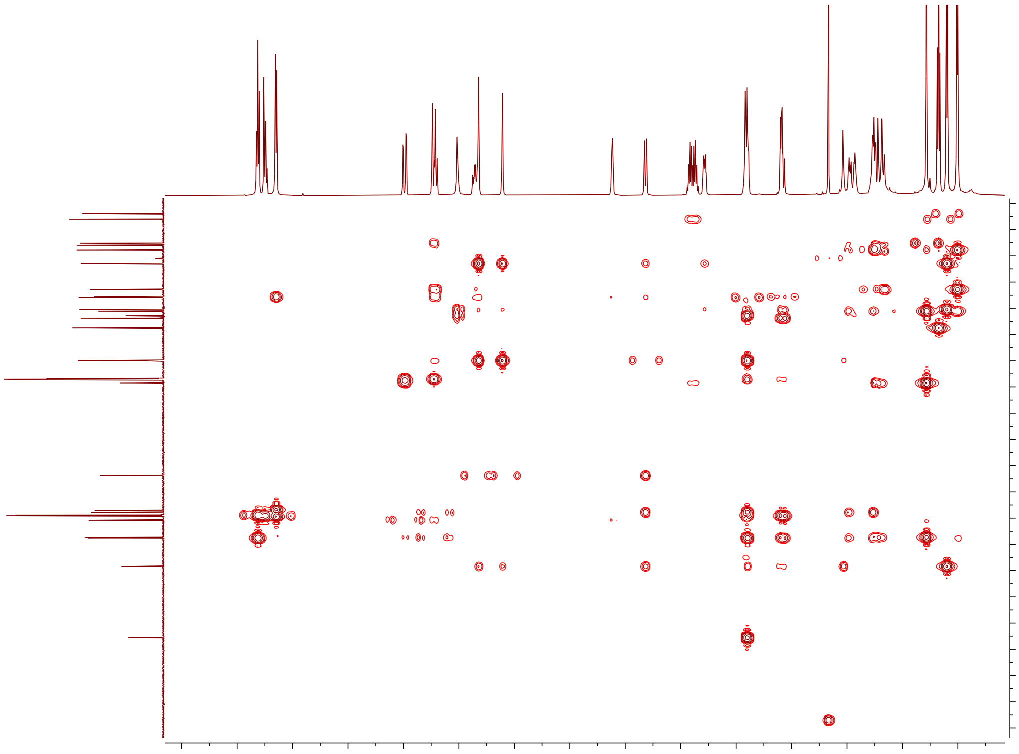

Figure S34. HMBC spectrum of **4** (600 MHz, $CDCl_3$).

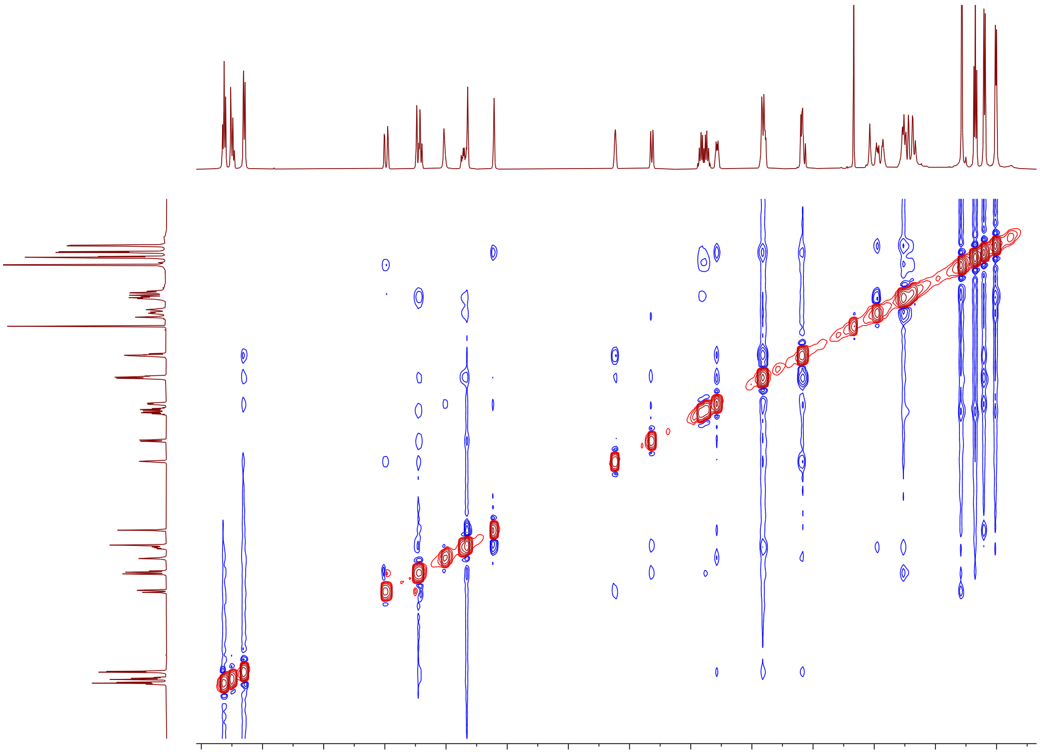

Figure S35. ROESY spectrum of **4** (600 MHz, $CDCl_3$).

Qualitative Analysis Report

**Data Filename**	SHJ13.d	**Sample Name**	SHJ13
**Sample Type**	Sample	**Position**	P1-A2
**Instrument Name**	Instrument 1	**User Name**	
**Acq Method**	SIBU.m	**Acquired Time**	12/25/2014 3:10:10 PM
**IRM Calibration Status**	Success	**DA Method**	Default.m
**Comment**			

Sample Group **Info.**
Acquisition SW 6200 series TOF/6500 series
Version Q-TOF B.05.01 (B5125.2)

User Spectra

Fragmentor Voltage	Collision Energy	Ionization Mode
135	0	ESI

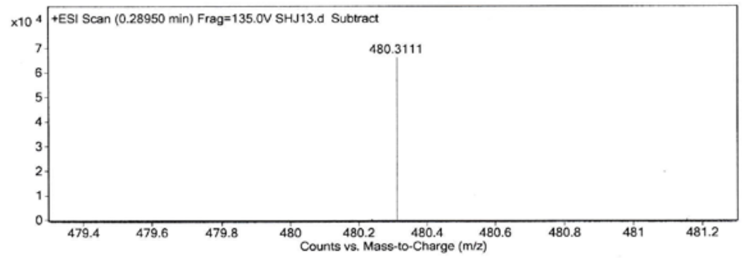

Peak List

m/z	z	Abund	Formula	Ion
434.2692	1	20036.47		
480.3111	1	66189.3	C30 H41 N O4	(M+H)+
502.2929	1	45060.74		
981.5964	1	33772.45		
982.6006	1	21777.8		

Formula Calculator Element Limits

Element	Min	Max
C	3	60
H	0	120
O	0	30
N	0	5

Formula Calculator Results

Formula	CalculatedMass	CalculatedMz	Mz	Diff. (mDa)	Diff. (ppm)	DBE
C30 H41 N O4	479.3036	480.3108	480.3111	-0.2	-0.5	11.0000

--- End Of Report ---

Agilent Technologies Page 1 of 1 Printed at: 3:22 PM on: 12/25/2014

Figure S36. HRESIMS spectrum of **4**.

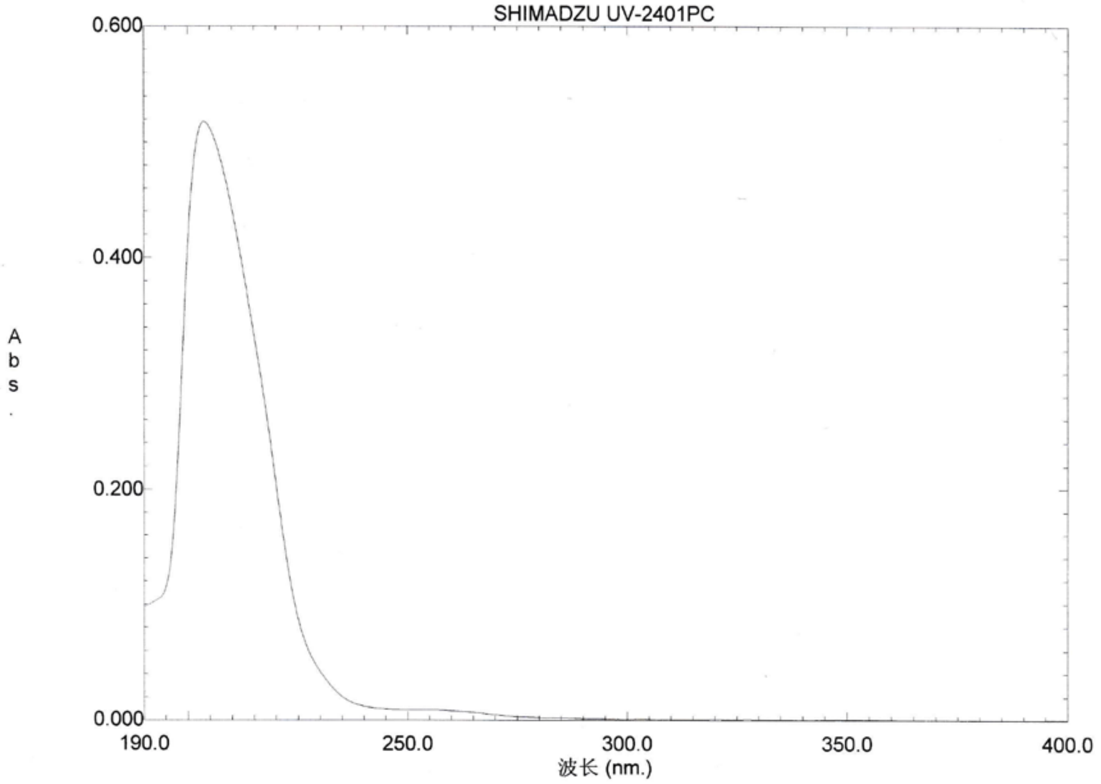

文件名: SHJ13

SHJ13 ———

样品浓度：0.0086毫克/毫升
溶剂：甲醇

创建于: 16:09 15-01-21
数据: 原始

测量模式: Abs.
扫描速度: 中速
狭缝: 5.0
采样间隔: 0.2

否.	波长 (nm.)	Abs.
1	203.80	0.5179

Figure S37. UV spectrum of **4**.

Optical rotation measurement

Model : P-1020 (A060460638)

No.	Sample	Mode	Data	Monitor<br>Blank	Temp.<br>Cell<br>Temp Point	Date<br>Comment<br>Sample Name	Light<br>Filter<br>Operator	Cycle Time<br>Integ Time
No.1	18 (1/3)	Sp.Rot	49.1100	0.0469<br>0.0000	18.2<br>50.00<br>Cell	Tue Jan 20 21:11:24 2015<br>0.00191g/mL MeOH<br>SHJ13	Na<br>589nm	2 sec<br>10 sec
No.2	18 (2/3)	Sp.Rot	50.1570	0.0479<br>0.0000	18.2<br>50.00<br>Cell	Tue Jan 20 21:11:37 2015<br>0.00191g/mL MeOH<br>SHJ13	Na<br>589nm	2 sec<br>10 sec
No.3	18 (3/3)	Sp.Rot	50.6810	0.0484<br>0.0000	18.2<br>50.00<br>Cell	Tue Jan 20 21:11:51 2015<br>0.00191g/mL MeOH<br>SHJ13	Na<br>589nm	2 sec<br>10 sec

+49.9835°

Figure S38. ORD spectrum of **4**.

SHJ13

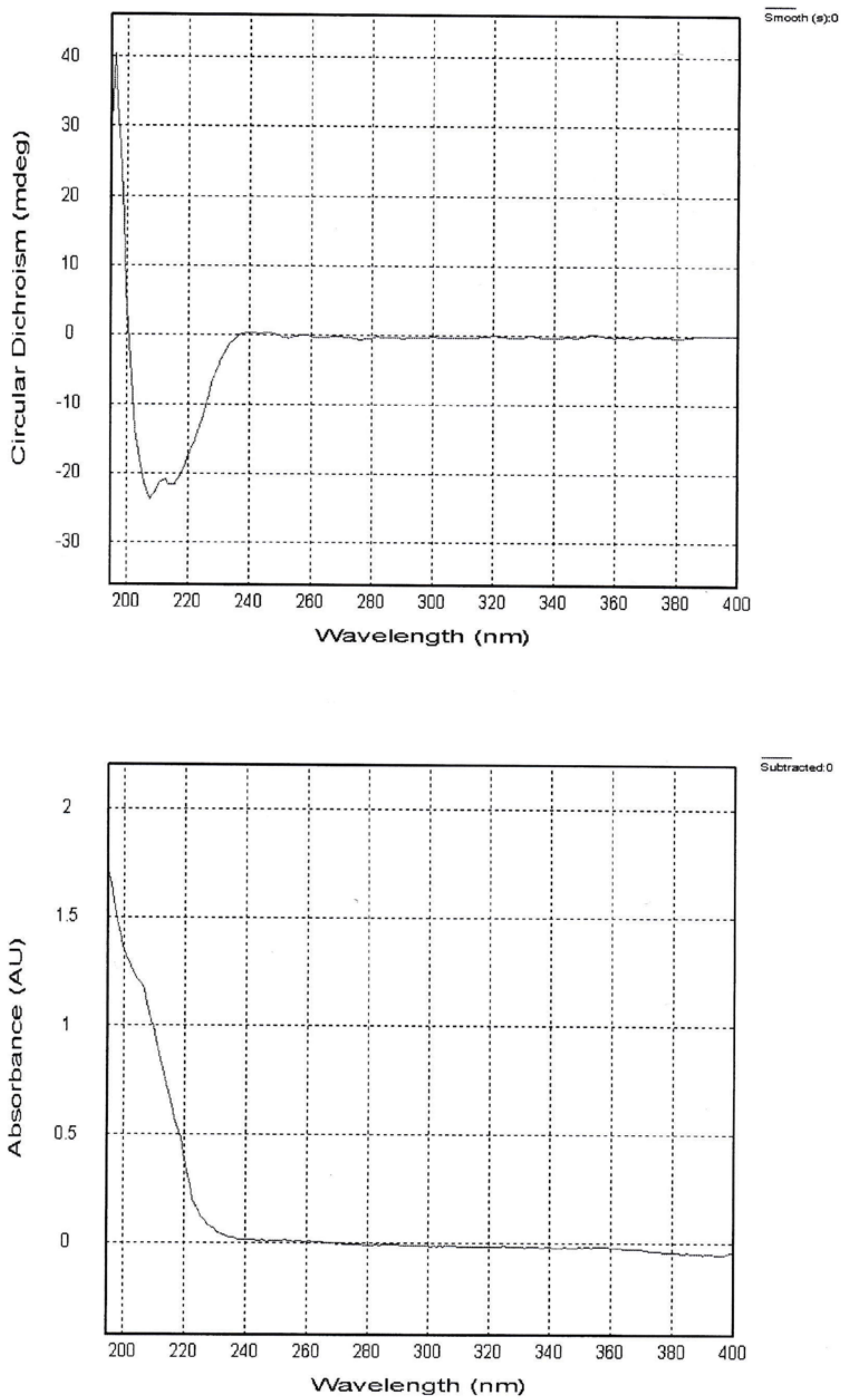

Figure S39. CD spectrum of **4**.

5. NMR, HRESIMS, UV, ORD, and CD spectra of compound 5

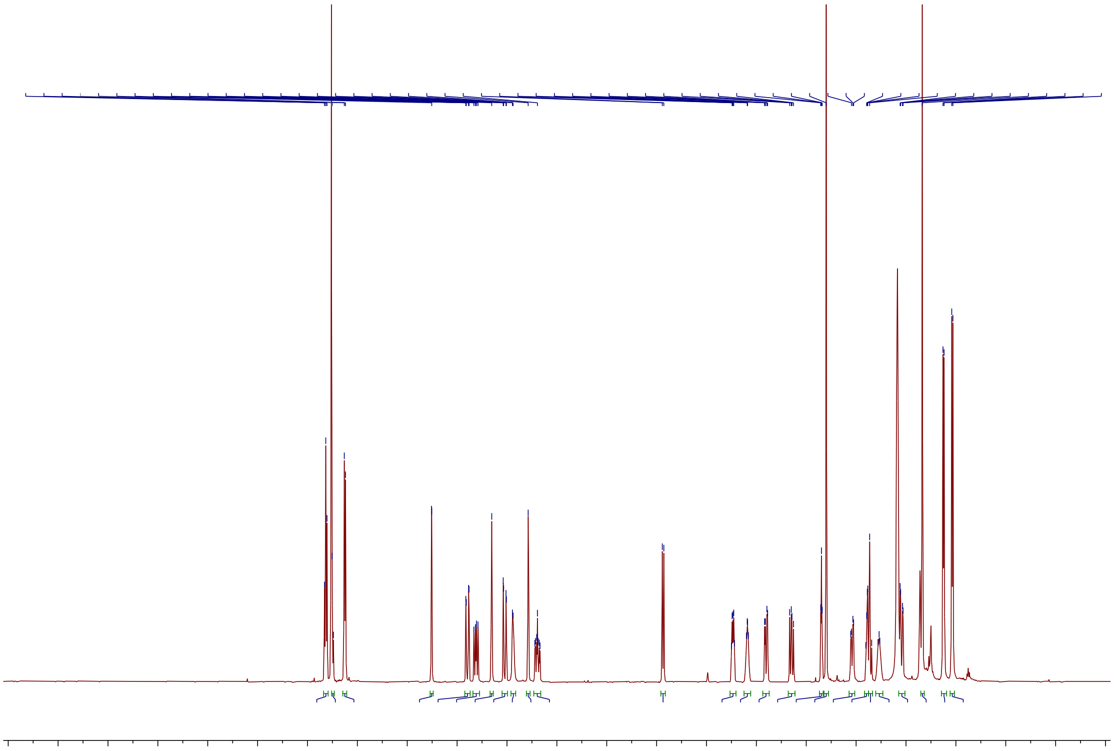

Figure S40. ^{1}H NMR spectrum of **5** (600 MHz, $CDCl_3$).

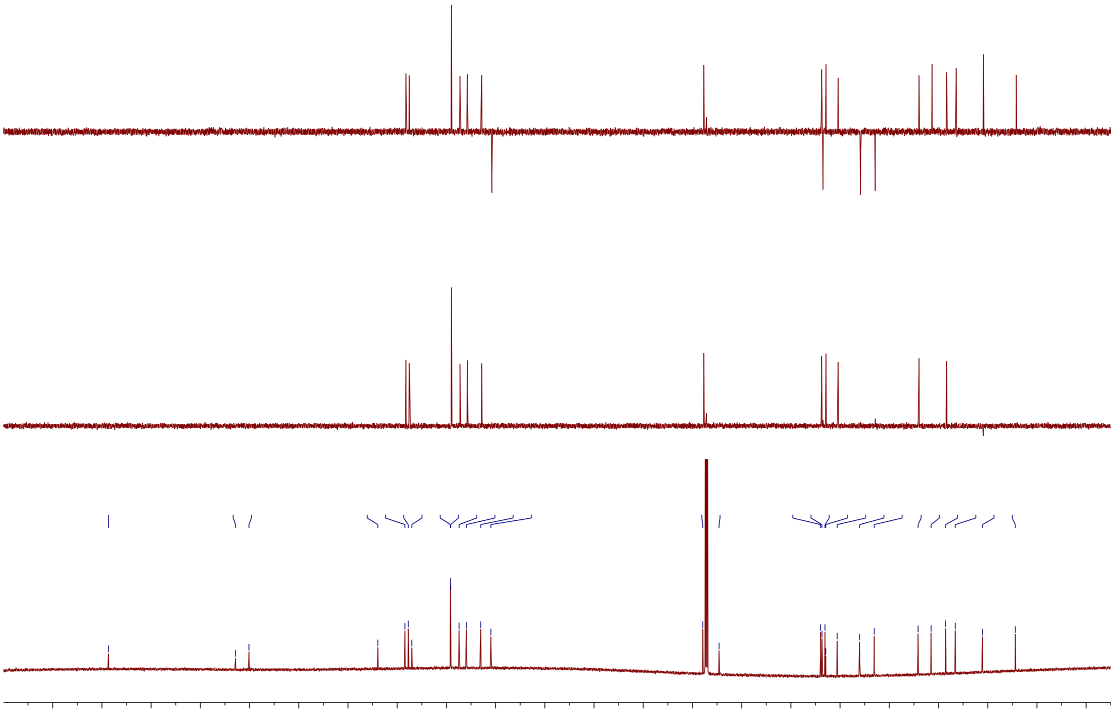

Figure S41. ^{13}C NMR, DEPT-90 and DEPT-135 spectra of **5** (150 MHz, $CDCl_3$).

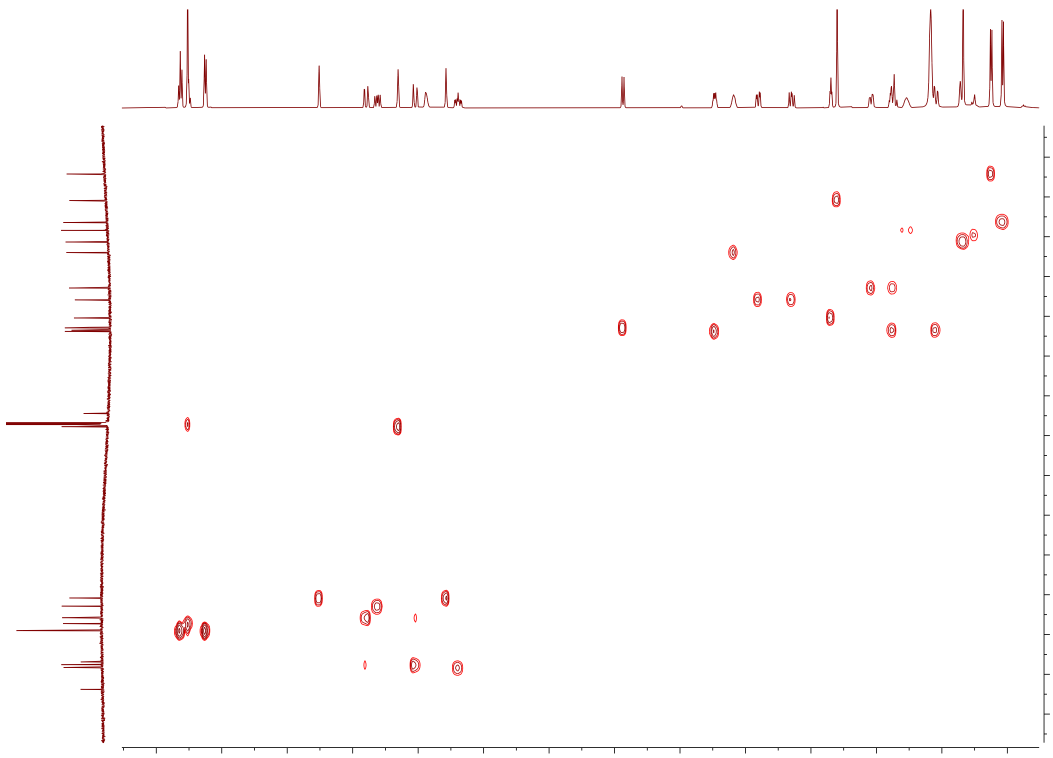

Figure S42. HSQC spectrum of **5** (600 MHz, $CDCl_3$).

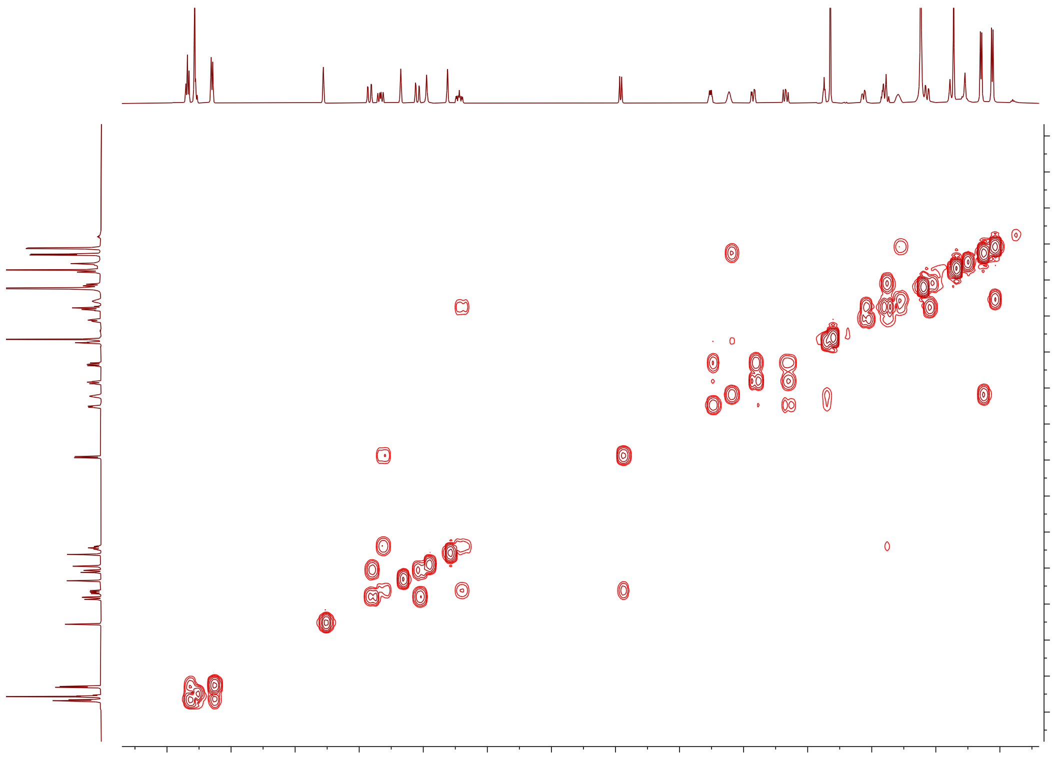

Figure S43. ^1H-^1H COSY spectrum of **5** (600 MHz, $CDCl_3$).

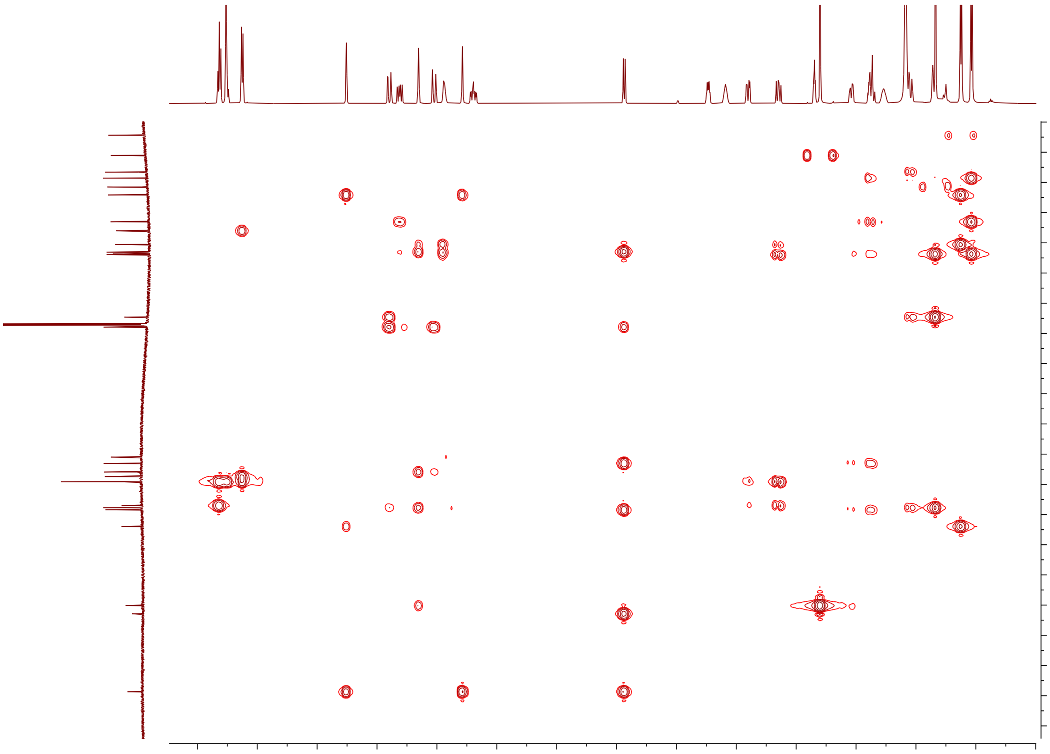

Figure S44. HMBC spectrum of **5** (600 MHz, $CDCl_3$).

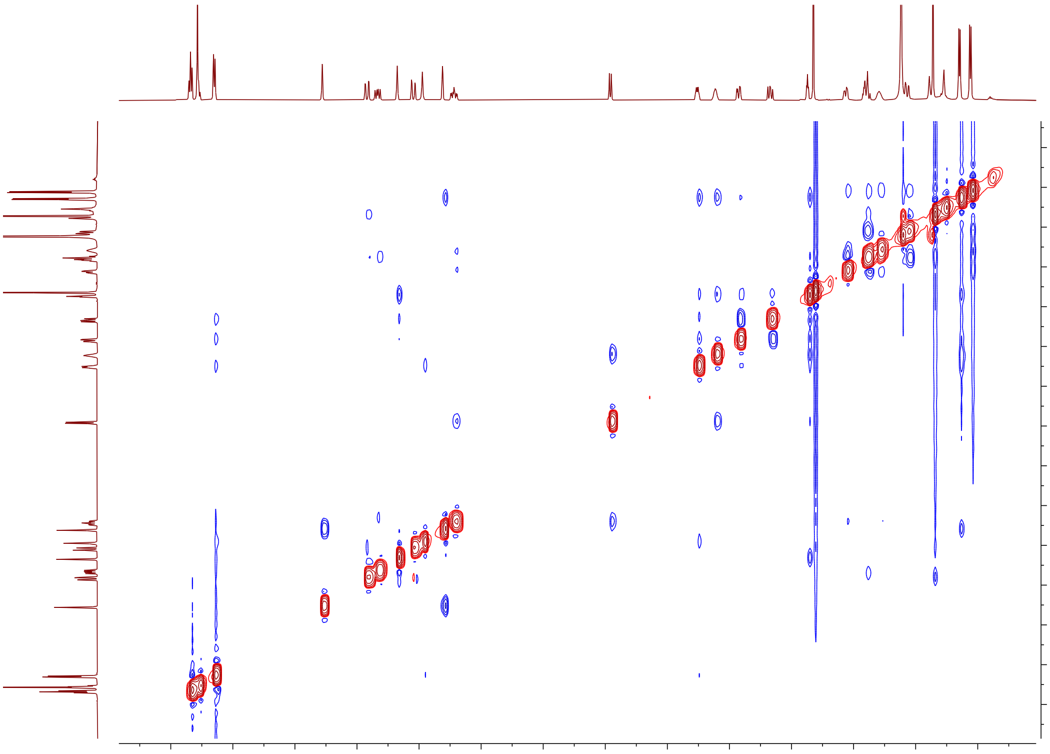

Figure S45. ROESY spectrum of **5** (600 MHz, $CDCl_3$).

Qualitative Analysis Report

**Data Filename**	SHJ29.d	**Sample Name**	SHJ29
**Sample Type**	Sample	**Position**	P1-A1
**Instrument Name**	Instrument 1	**User Name**	
**Acq Method**	SIBU.m	**Acquired Time**	7/16/2015 3:13:22 PM
**IRM Calibration Status**	Success	**DA Method**	Default.m
**Comment**			

Sample Group **Info.**
Acquisition SW Version 6200 series TOF/6500 series Q-TOF B.05.01 (B5125.2)

User Spectra

Fragmentor Voltage	Collision Energy	Ionization Mode
135	0	ESI

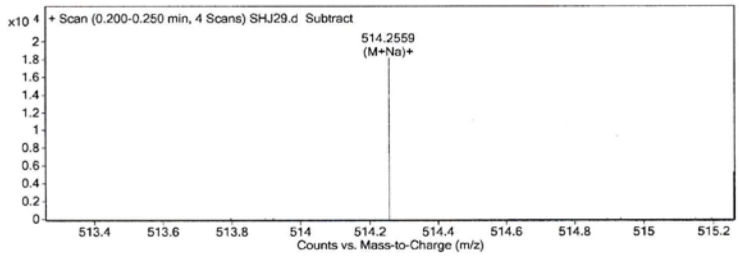

Peak List

m/z	z	Abund	Formula	Ion
102.128		2445.02		
306.6404	2	2378.82		
432.2536	1	3563.13		
492.2744	1	4787.3		
514.2559	1	18229.21	C30 H37 N O5	(M+Na)+
515.2594	1	5280.5	C30 H37 N O5	(M+Na)+
530.2295	1	7563.51		
531.2326	1	2451		

Formula Calculator Element Limits

Element	Min	Max
C	3	60
H	0	120
O	0	10
N	0	5

Formula Calculator Results

Formula	CalculatedMass	CalculatedMz	Mz	Diff. (mDa)	Diff. (ppm)	DBE
C30 H37 N O5	491.2672	514.2564	514.2559	0.4	0.9	13.0000

--- End Of Report ---

Agilent Technologies Page 1 of 1 Printed at: 3:17 PM on: 7/16/2015

Figure S46. HRESIMS spectrum of **5**.

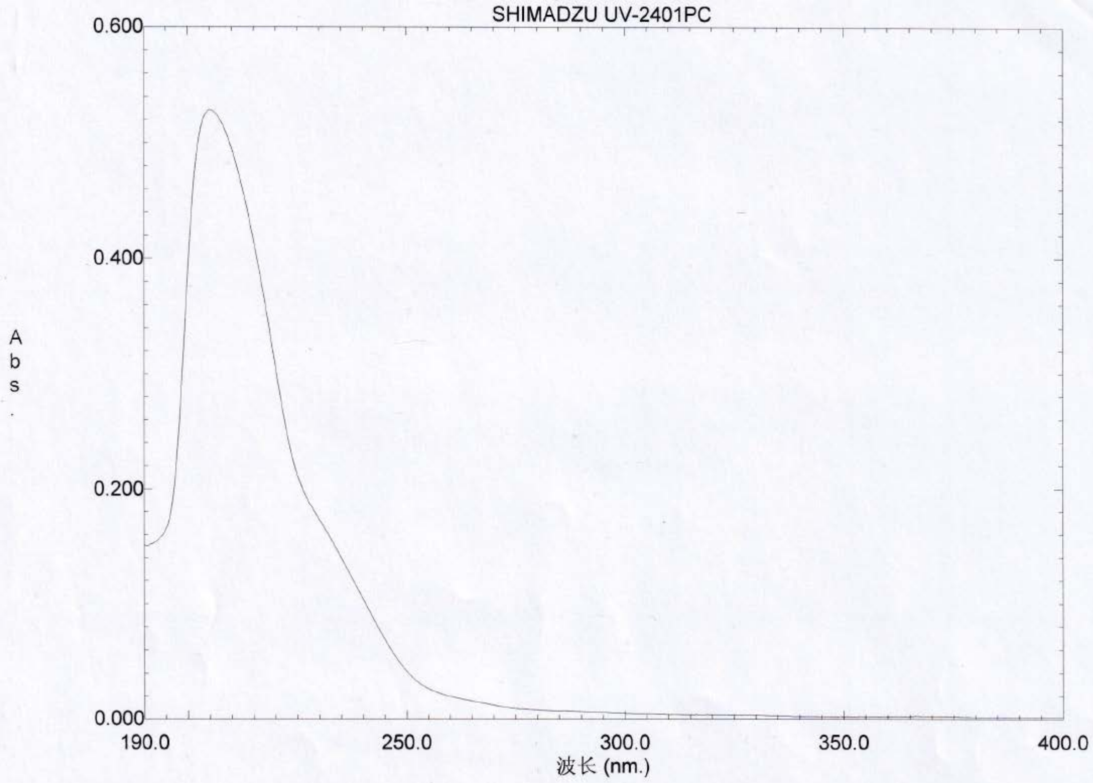

文件名: SHJX29

样品浓度：0.0122毫克/毫升
溶剂：甲醇

创建于: 14:57 15-08-10
数据: 原始

测量模式: Abs.
扫描速度: 中速
狭缝: 5.0
采样间隔: 0.5

否.	波长 (nm.)	Abs.
1	445.00	0.0024
2	414.00	0.0028
3	205.00	0.5284

Figure S47. UV spectrum of **5**.

Optical rotation measurement

Model : P-1020 (A060460638)

No.	Sample	Mode	Data	Monitor<br>Blank	Temp.<br>Cell<br>Temp Point	Date<br>Comment<br>Sample Name	Light<br>Filter<br>Operator	Cycle Time<br>Integ Time	
No.1	13 (1/3)	Sp.Rot	-11.6840	-0.0111<br>0.0000	23.6<br>50.00<br>Cell	Thu Aug 06 17:34:16 2015<br>0.00190g/mL MeOH<br>SHJX29	Na<br>589nm	2 sec<br>10 sec	
No.2	13 (2/3)	Sp.Rot	-12.9470	-0.0123<br>0.0000	23.5<br>50.00<br>Cell	Thu Aug 06 17:34:29 2015<br>0.00190g/mL MeOH<br>SHJX29	Na<br>589nm	2 sec<br>10 sec	-12.2807°
No.3	13 (3/3)	Sp.Rot	-12.2110	-0.0116<br>0.0000	23.4<br>50.00<br>Cell	Thu Aug 06 17:34:43 2015<br>0.00190g/mL MeOH<br>SHJX29	Na<br>589nm	2 sec<br>10 sec	

Figure S48. ORD spectrum of **5**.

SHJX29

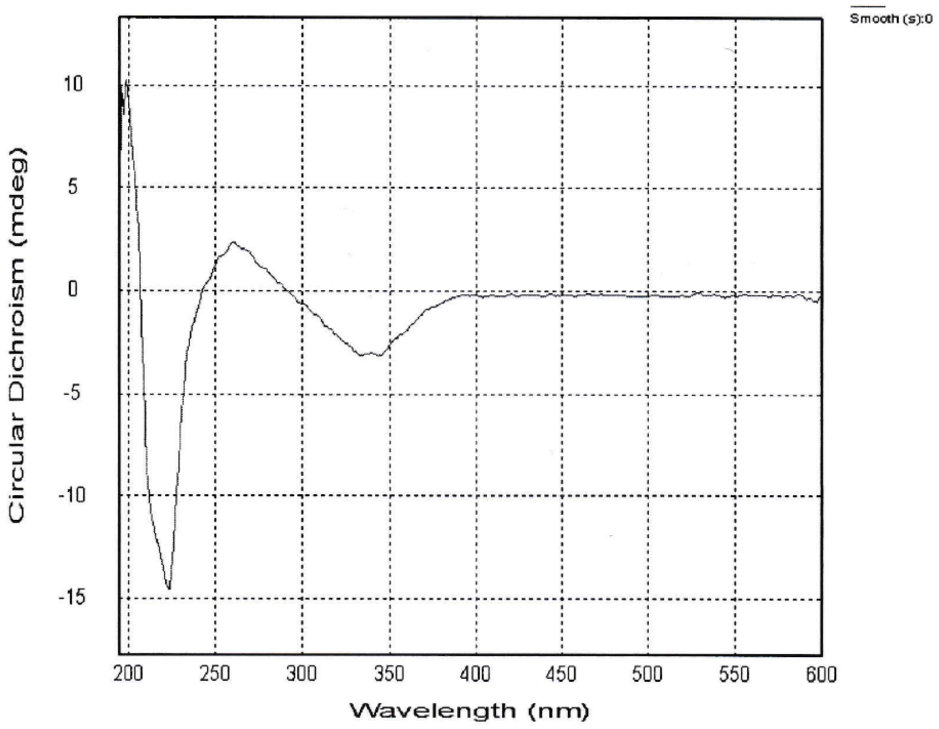

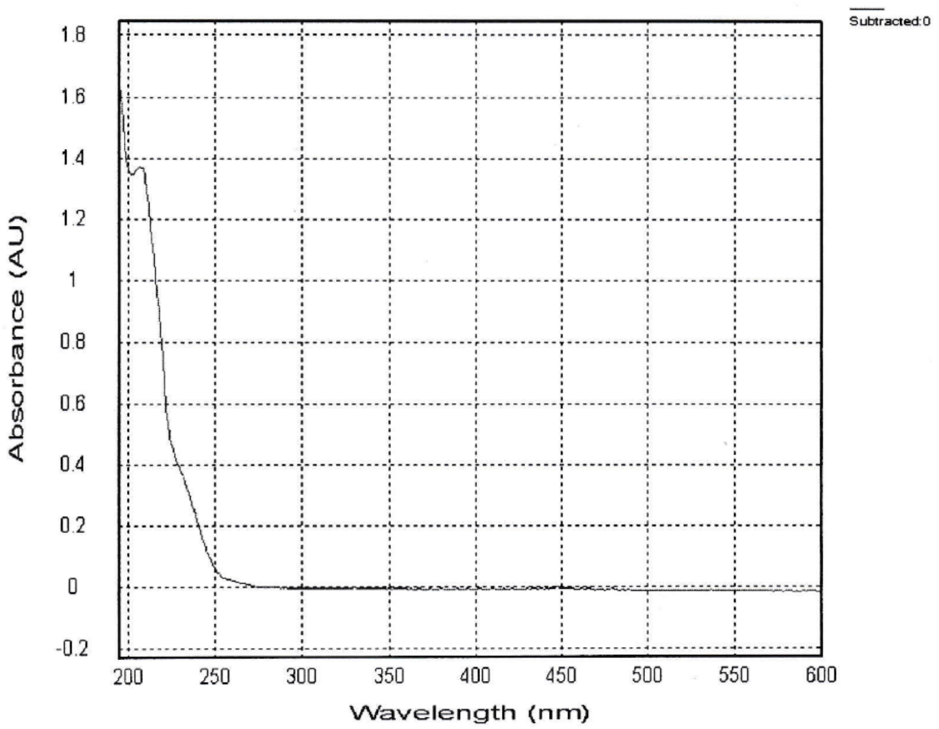

Figure S49. CD spectrum of **5**.

6. NMR, HRESIMS, UV, ORD, and CD spectra of compound 6

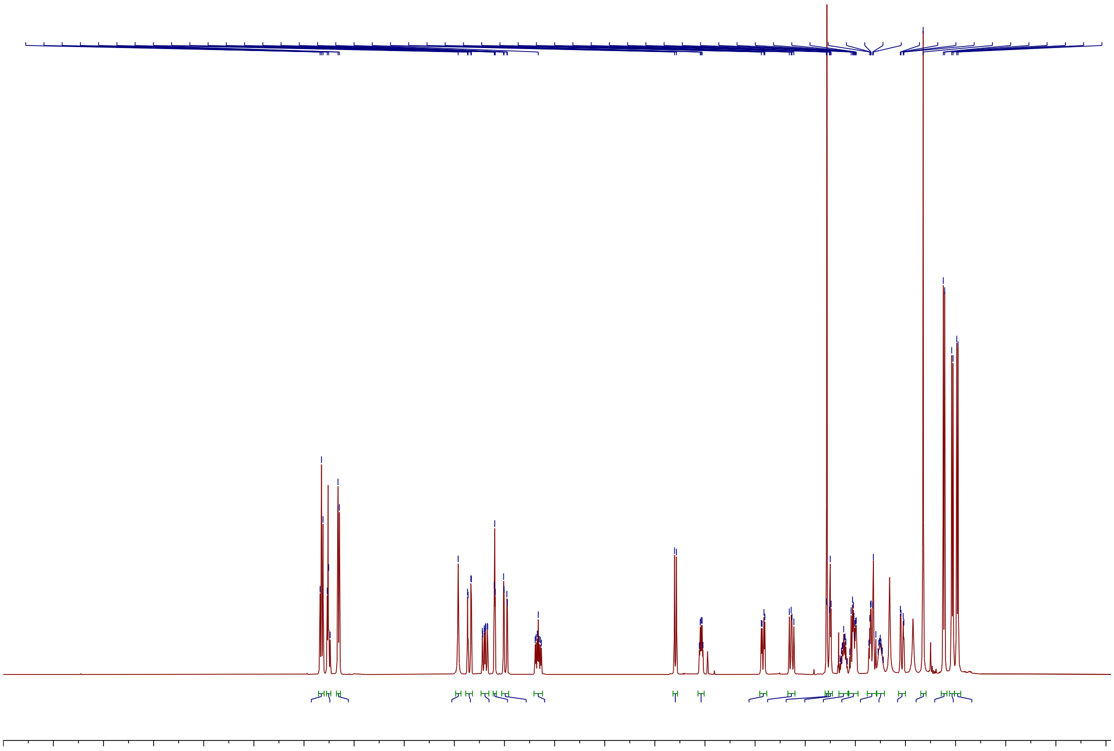

Figure S50. ^{1}H NMR spectrum of **6** (500 MHz, $CDCl_3$).

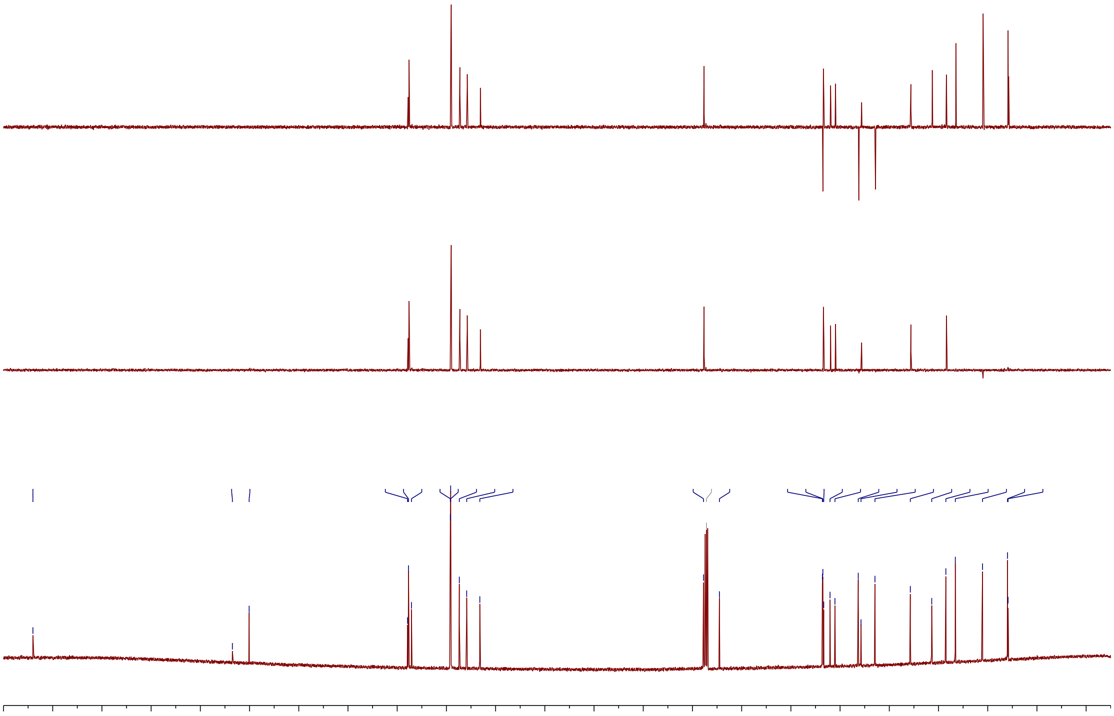

Figure S51. ^{13}C NMR, DEPT-90 and DEPT-135 spectra of **6** (125 MHz, $CDCl_3$).

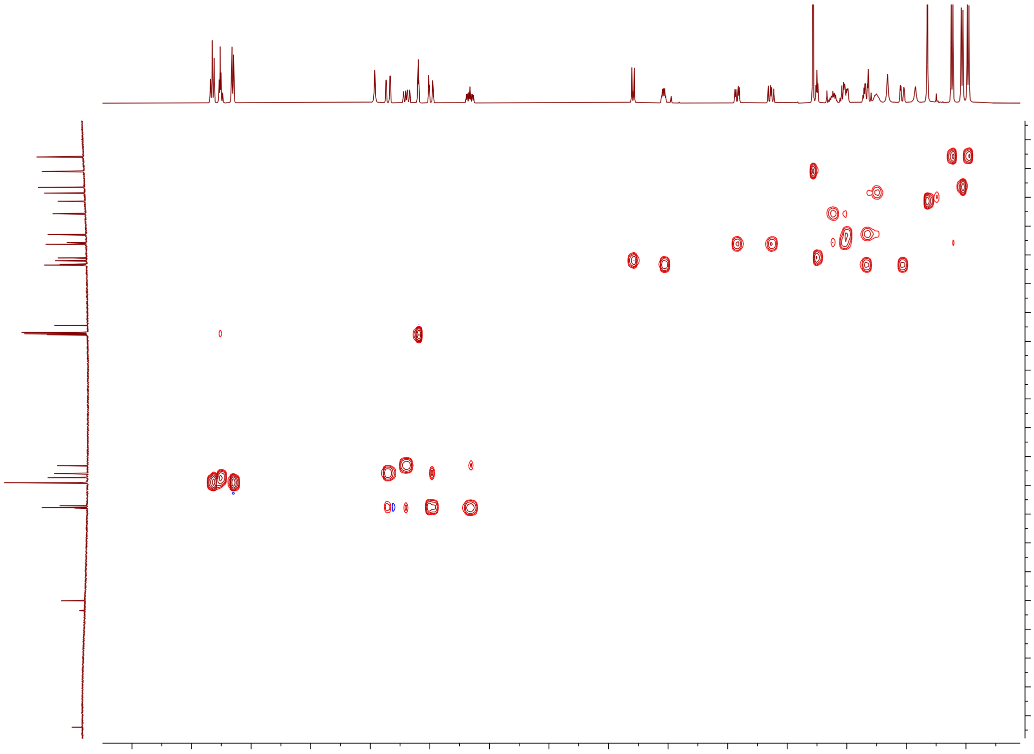

Figure S52. HSQC spectrum of **6** (500 MHz, $CDCl_3$).

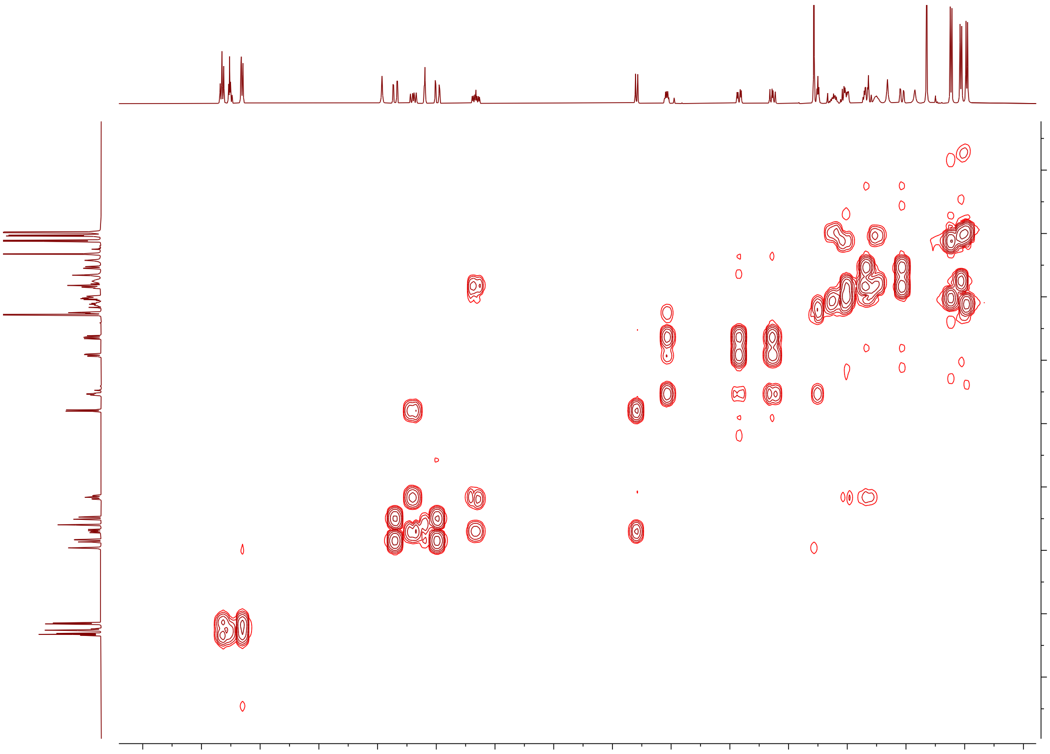

Figure S53. ^1H-^1H COSY spectrum of **6** (500 MHz, $CDCl_3$).

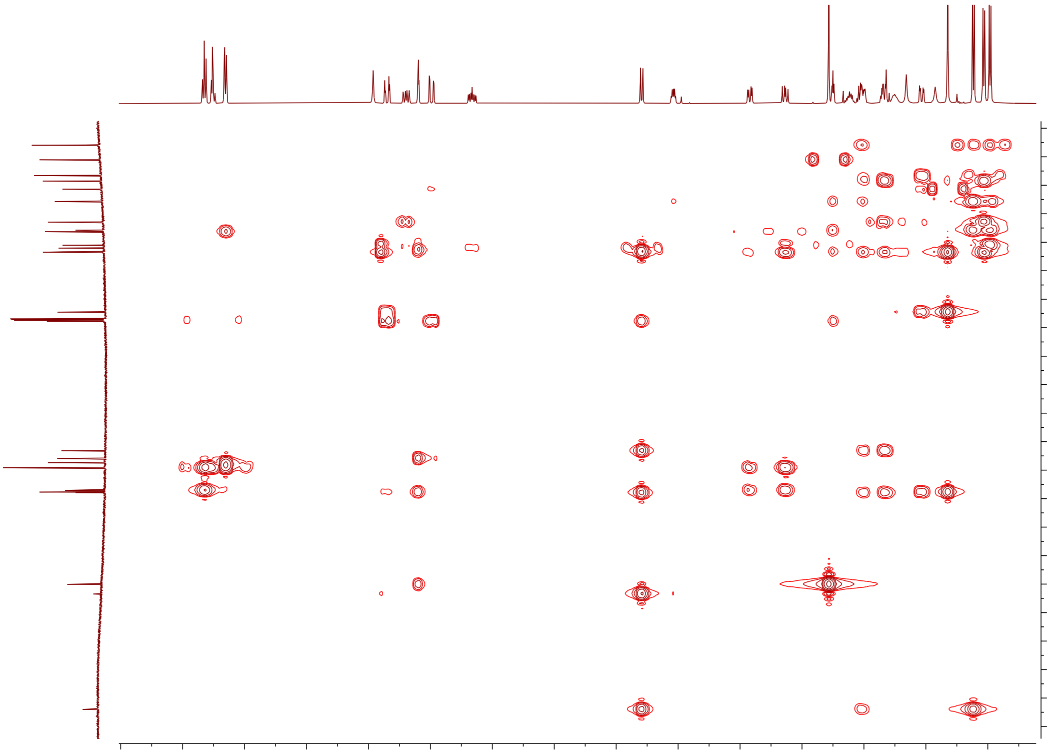

Figure S54. HMBC spectrum of **6** (500 MHz, $CDCl_3$).

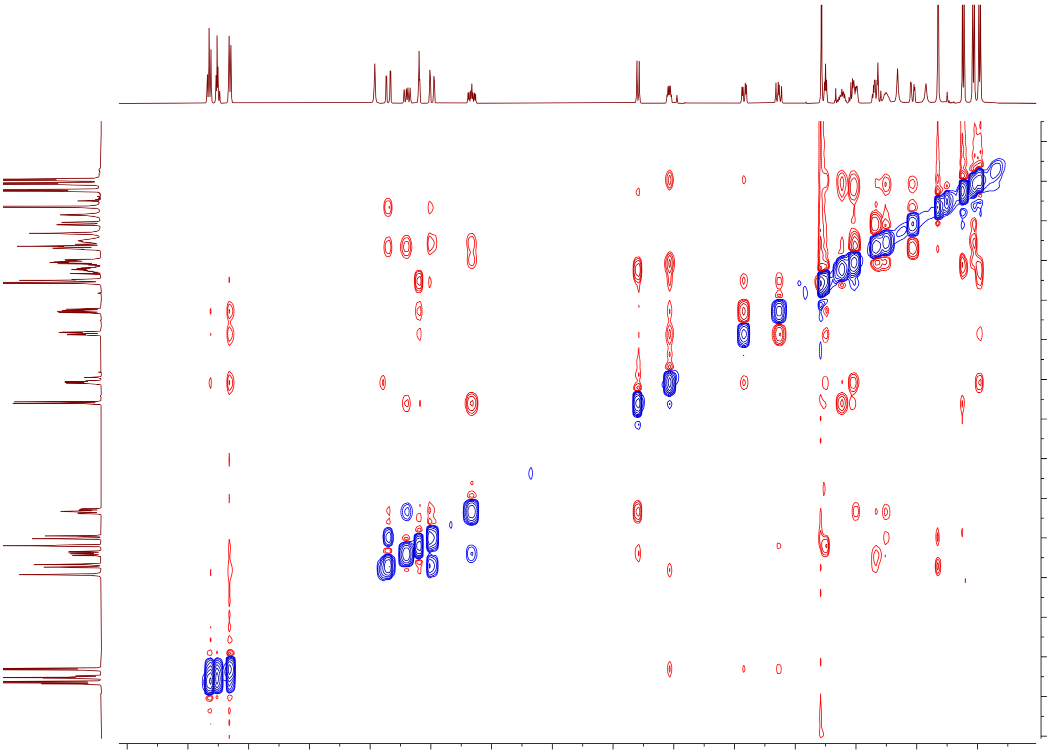

Figure S55. ROESY spectrum of **6** (500 MHz, $CDCl_3$).

Qualitative Analysis Report

**Data Filename**	SHJ31.d	**Sample Name**	SHJ31
**Sample Type**	Sample	**Position**	P1-A2
**Instrument Name**	Instrument 1	**User Name**	
**Acq Method**	SIBU.m	**Acquired Time**	7/16/2015 3:14:52 PM
**IRM Calibration Status**	Success	**DA Method**	Default.m
**Comment**			

Sample Group **Info.**

Acquisition SW Version 6200 series TOF/6500 series Q-TOF B.05.01 (B5125.2)

User Spectra

Fragmentor Voltage	Collision Energy	Ionization Mode
135	0	ESI

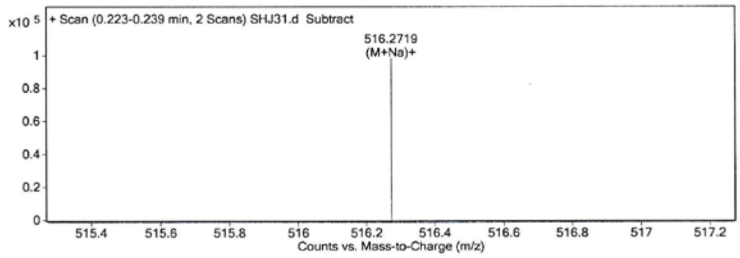

Peak List

m/z	z	Abund	Formula	Ion
494.2902	1	34874.45		
516.2719	1	98321.98	C30 H39 N O5	(M+Na)+
532.245	1	40913.93		
987.5729	1	45465.29		
1009.5546	1	49088.25		
1025.5275	1	37422.34		
1502.8366	1	59278.76		
1503.8395	1	61104.78		

Formula Calculator Element Limits

Element	Min	Max
C	3	60
H	0	120
O	0	10
N	0	5

Formula Calculator Results

Formula	CalculatedMass	CalculatedMz	Mz	Diff. (mDa)	Diff. (ppm)	DBE
C30 H39 N O5	493.2828	516.2720	516.2719	0.3	0.5	12.0000

--- End Of Report ---

Agilent Technologies Page 1 of 1 Printed at: 3:17 PM on: 7/16/2015

Figure S56. HRESIMS spectrum of **6**.

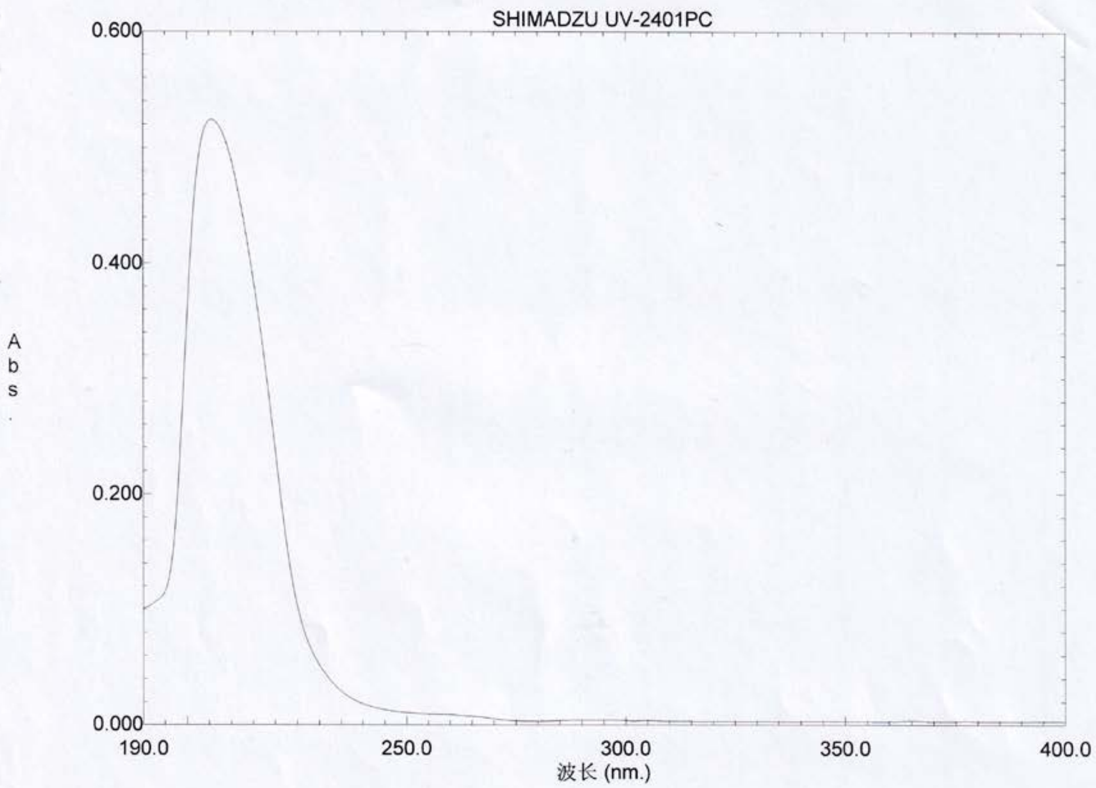

文件名: SHJX31

SHJX31 ———

样品浓度：0.0108毫克/毫升
溶剂：甲醇

创建于: 15:02 15-08-10
数据: 原始

测量模式: Abs.
扫描速度: 中速
狭缝: 5.0
采样间隔: 0.5

否.	波长 (nm.)	Abs.
1	514.50	0.0044
2	441.00	0.0040
3	205.50	0.5241

Figure S57. UV spectrum of **6**.

Optical rotation measurement

Model : P-1020 (A060460638)

No.	Sample	Mode	Data	Monitor<br>Blank	Temp.<br>Cell<br>Temp Point	Date<br>Comment<br>Sample Name	Light<br>Filter<br>Operator	Cycle Time<br>Integ Time	
No.1	14 (1/3)	Sp.Rot	-64.2490	-0.0620<br>0.0000	23.6<br>50.00<br>Cell	Thu Aug 06 17:42:47 2015<br>0.00193g/mL MeOH<br>SHJX31	Na<br>589nm	2 sec<br>10 sec	
No.2	14 (2/3)	Sp.Rot	-63.1090	-0.0609<br>0.0000	23.6<br>50.00<br>Cell	Thu Aug 06 17:43:00 2015<br>0.00193g/mL MeOH<br>SHJX31	Na<br>589nm	2 sec<br>10 sec	-63.1779°
No.3	14 (3/3)	Sp.Rot	-62.1760	-0.0600<br>0.0000	23.6<br>50.00<br>Cell	Thu Aug 06 17:43:14 2015<br>0.00193g/mL MeOH<br>SHJX31	Na<br>589nm	2 sec<br>10 sec	

Figure S58. ORD spectrum of **6**.

SHJX31

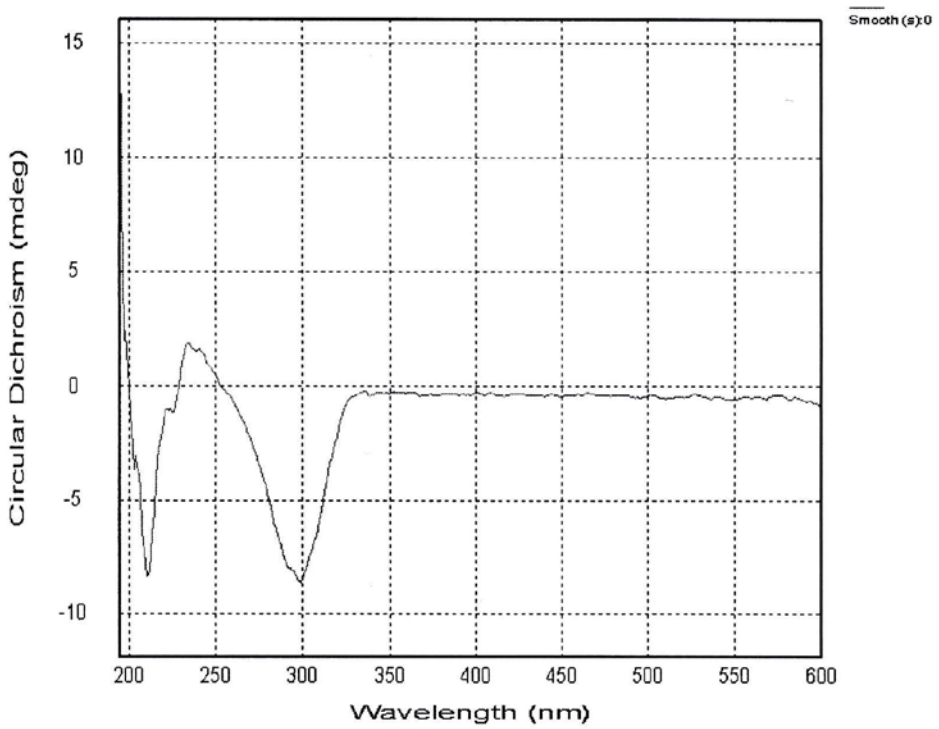

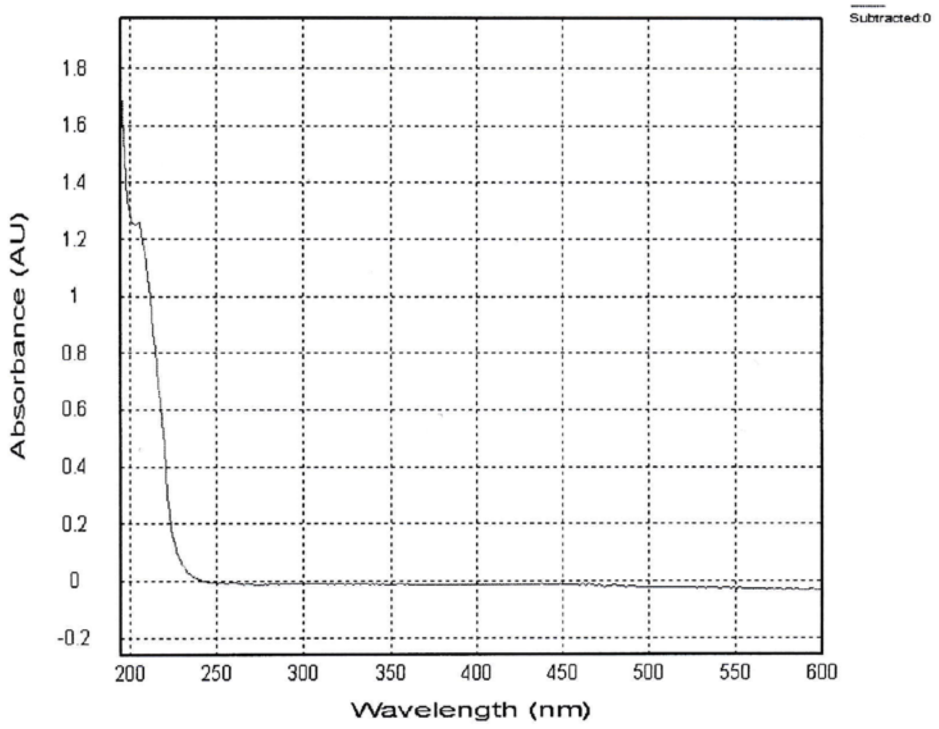

Figure S59. CD spectrum of **6**.

7. NMR, HRESIMS, UV, ORD, and CD spectra of compound 7

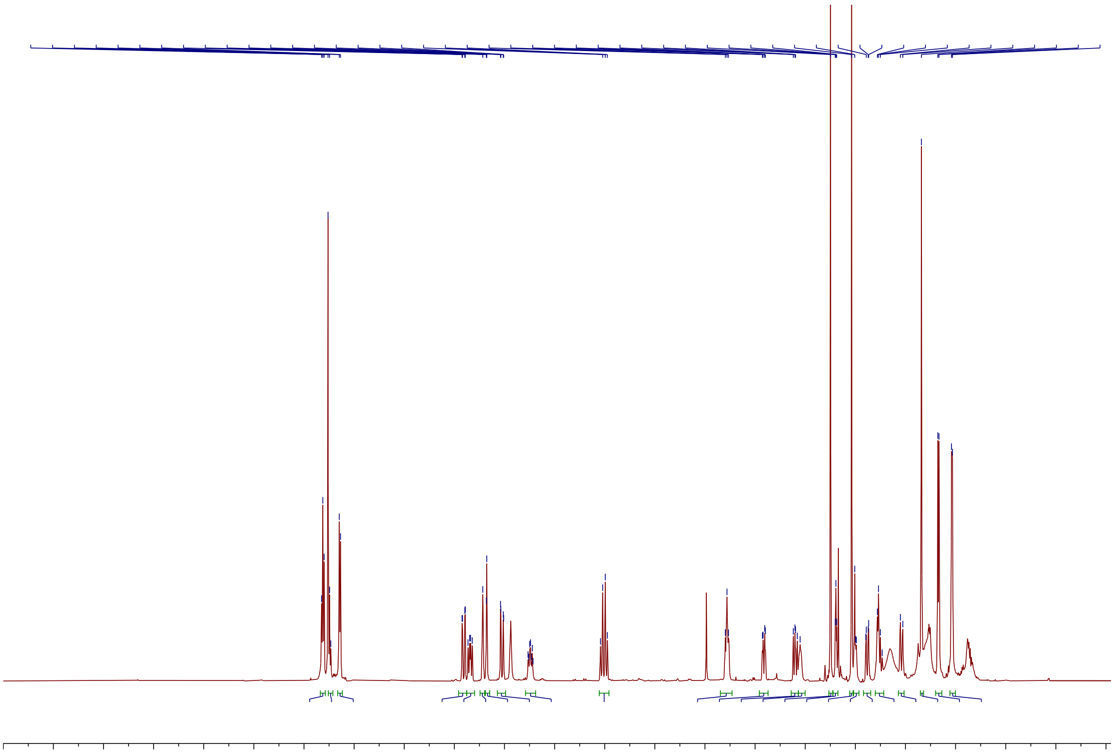

Figure S60. ^{1}H NMR spectrum of **7** (600 MHz, $CDCl_3$).

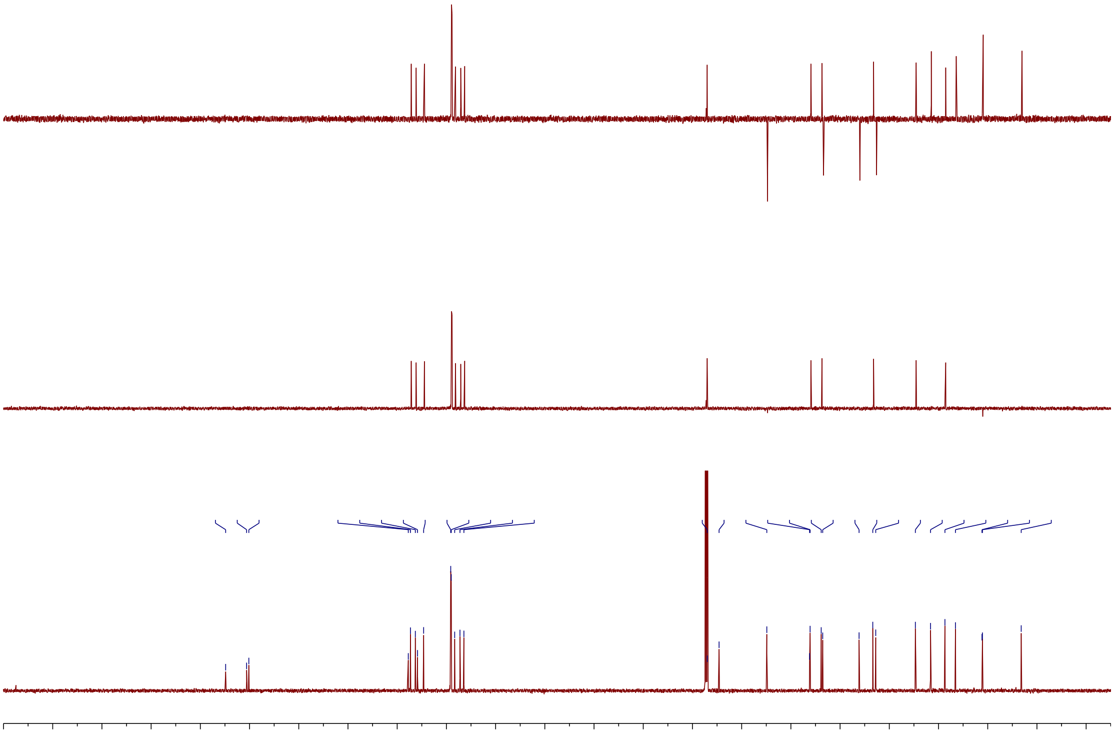

Figure S61. ^{13}C NMR, DEPT-90 and DEPT-135 spectra of **7** (150 MHz, $CDCl_3$).

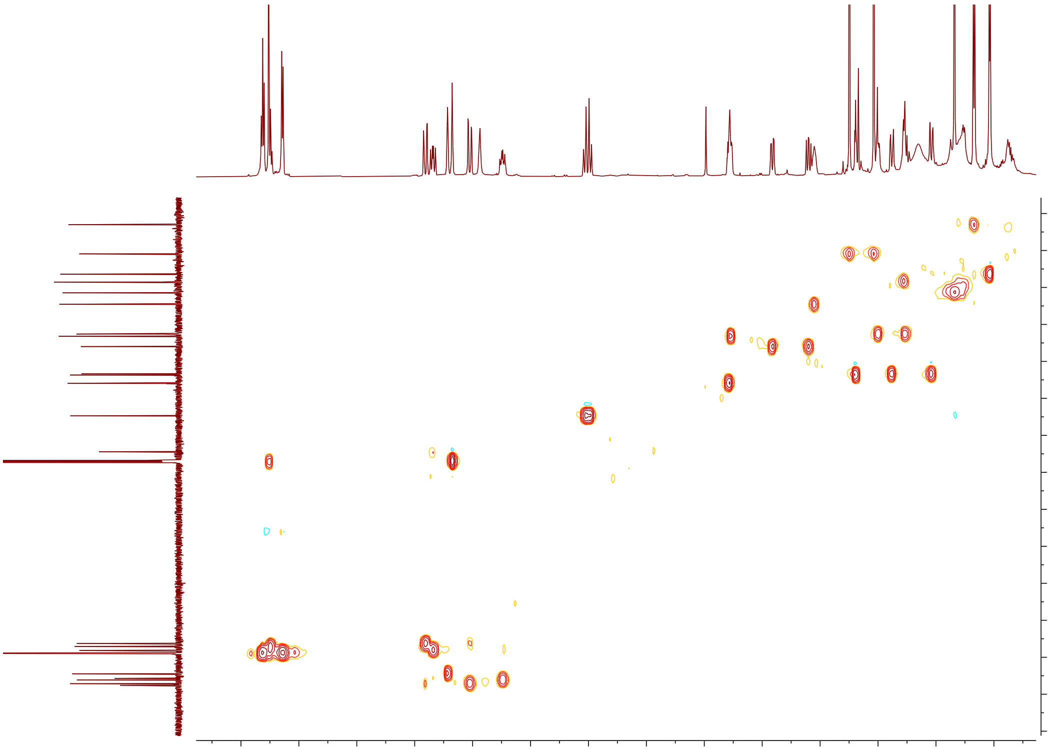

Figure S62. HSQC spectrum of **7** (600 MHz, $CDCl_3$).

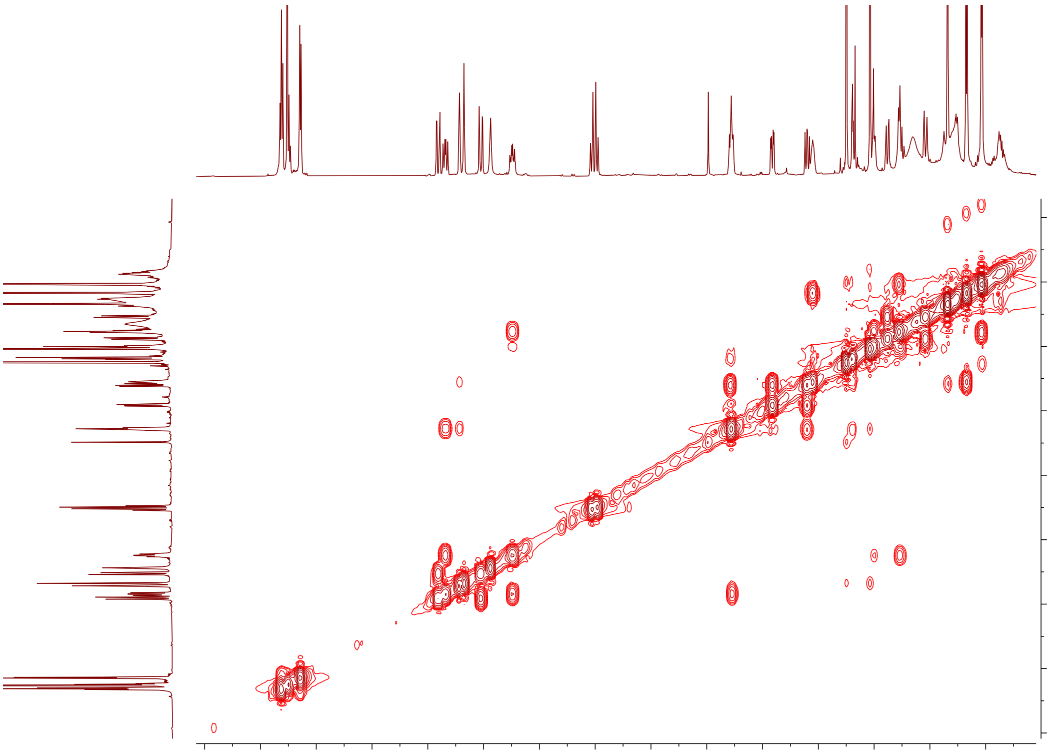

Figure S63. ^{1}H-^{1}H COSY spectrum of **7** (600 MHz, $CDCl_3$).

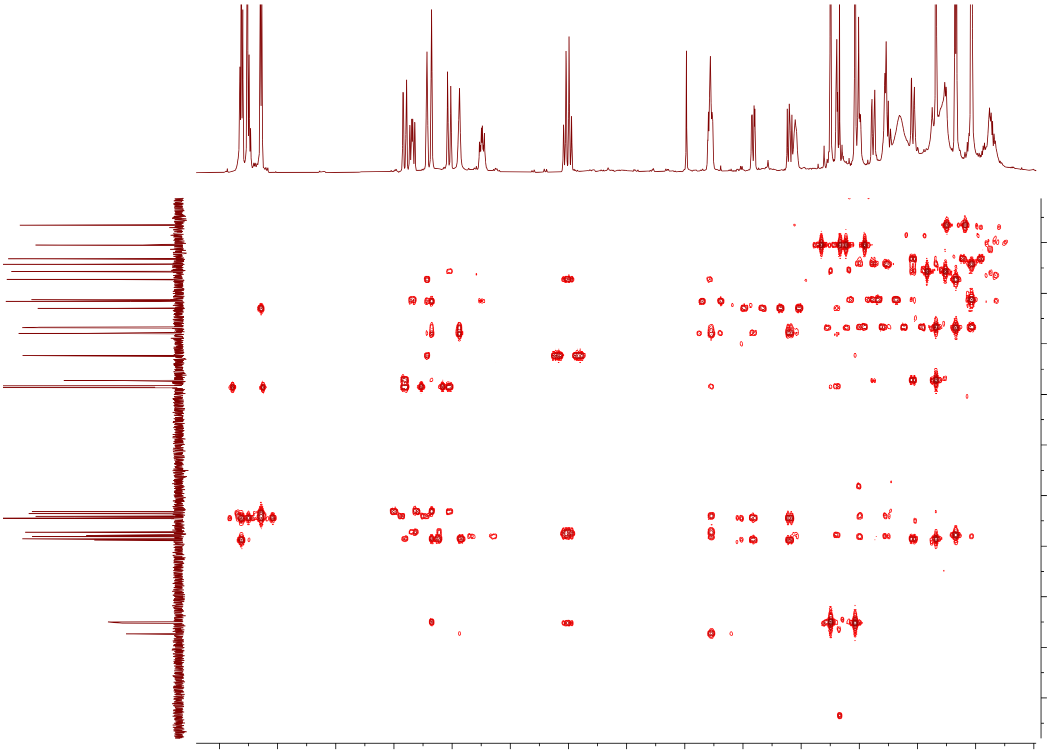

Figure S64. HMBC spectrum of **7** (600 MHz, $CDCl_3$).

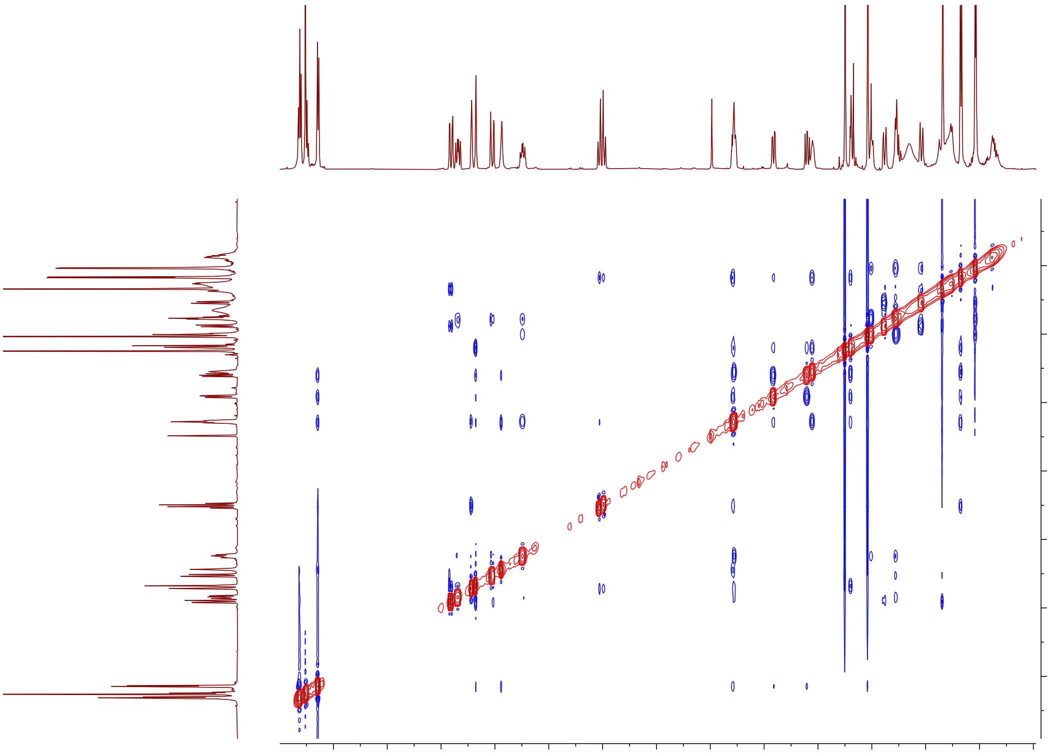

Figure S65. ROESY spectrum of **7** (600 MHz, $CDCl_3$).

Qualitative Analysis Report

**Data Filename**	SHJ20.d	**Sample Name**	SHJ20
**Sample Type**	Sample	**Position**	P1-A1
**Instrument Name**	Instrument 1	**User Name**	
**Acq Method**	SIBU.m	**Acquired Time**	12/30/2014 1:43:33 PM
**IRM Calibration Status**	Success	**DA Method**	Default.m
**Comment**			

Sample Group **Info.**

Acquisition SW Version 6200 series TOF/6500 series Q-TOF B.05.01 (B5125.2)

User Spectra

Fragmentor Voltage	Collision Energy	Ionization Mode
135	0	ESI

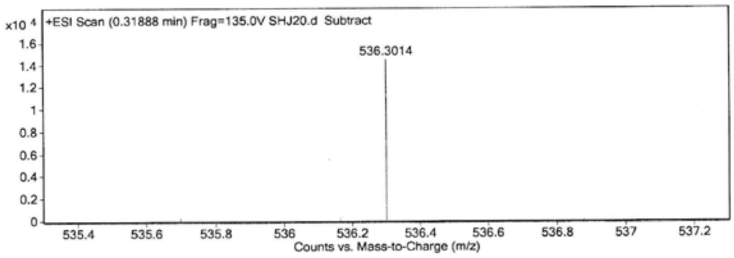

Peak List

m/z	z	Abund	Formula	Ion
416.2584	1	18167.43		
476.2792	1	37347.07		
536.3014	1	14513.92	C32 H41 N O6	(M+H)+
558.2831	1	73034.31		
559.2863	1	29266.66		

Formula Calculator Element Limits

Element	Min	Max
C	3	60
H	0	120
O	0	10
N	0	3

Formula Calculator Results

Formula	CalculatedMass	CalculatedMz	Mz	Diff. (mDa)	Diff. (ppm)	DBE
C32 H41 N O6	535.2934	536.3007	536.3014	-0.4	-0.8	13.0000

--- End Of Report ---

Agilent Technologies Page 1 of 1 Printed at: 1:53 PM on: 12/30/2014

Figure S66. HRESIMS spectrum of **7**.

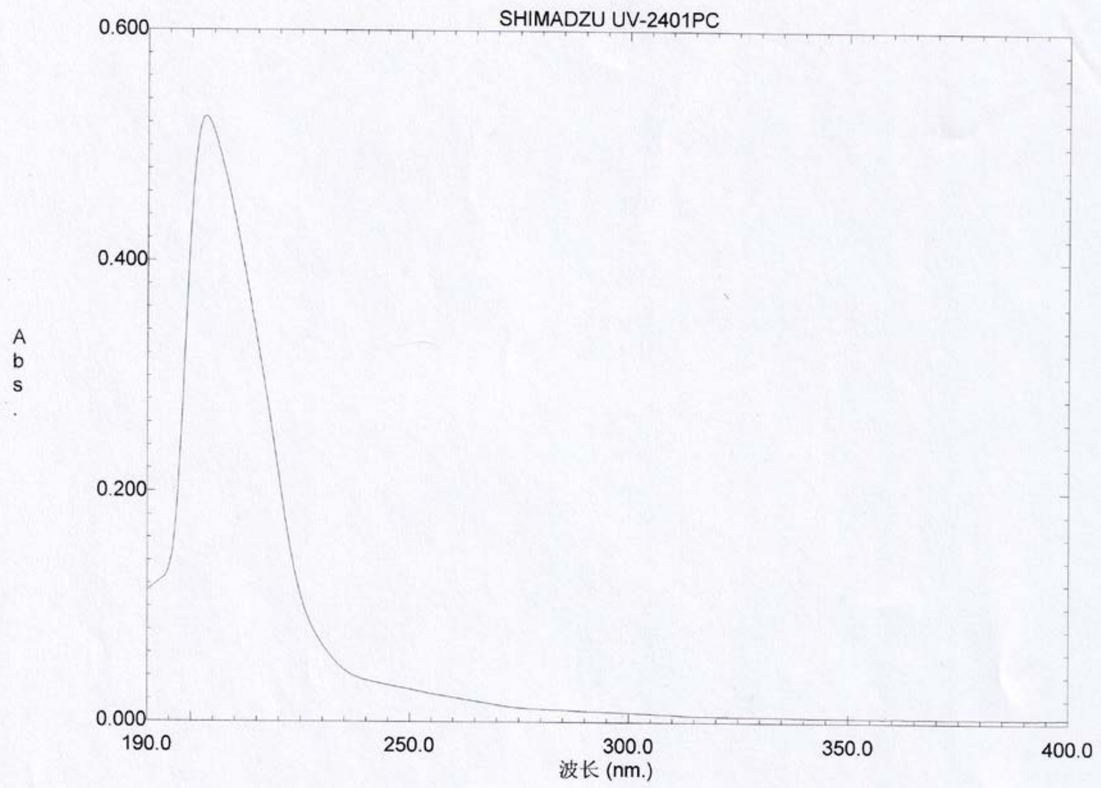

文件名: SHJ20

SHJ20 ———

样品浓度：0.0125毫克/毫升
溶剂：甲醇

创建于: 15:38 15-01-23
数据: 原始

测量模式: Abs.
扫描速度: 中速
狭缝: 5.0
采样间隔: 0.2

否.	波长 (nm.)	Abs.
1	203.20	0.5250

Figure S67. UV spectrum of **7**.

Optical rotation measurement

Model : P-1020 (A060460638)

No.	Sample	Mode	Data	Monitor<br>Blank	Temp.<br>Cell<br>Temp Point	Date<br>Comment<br>Sample Name	Light<br>Filter<br>Operator	Cycle Time<br>Integ Time
No.1	21 (1/3)	Sp.Rot	-28.5870	-0.0263<br>0.0000	19.6<br>50.00<br>Cell	Thu Jan 22 18:33:53 2015<br>0.00184g/mL MeOH<br>SHJ20	Na<br>589nm	2 sec<br>10 sec
No.2	21 (2/3)	Sp.Rot	-29.4570	-0.0271<br>0.0000	19.6<br>50.00<br>Cell	Thu Jan 22 18:34:06 2015<br>0.00184g/mL MeOH<br>SHJ20	Na<br>589nm	2 sec<br>10 sec
No.3	21 (3/3)	Sp.Rot	-27.1740	-0.0250<br>0.0000	19.6<br>50.00<br>Cell	Thu Jan 22 18:34:20 2015<br>0.00184g/mL MeOH<br>SHJ20	Na<br>589nm	2 sec<br>10 sec

-28.4058°

Figure S68. ORD spectrum of **7**.

SHJ20

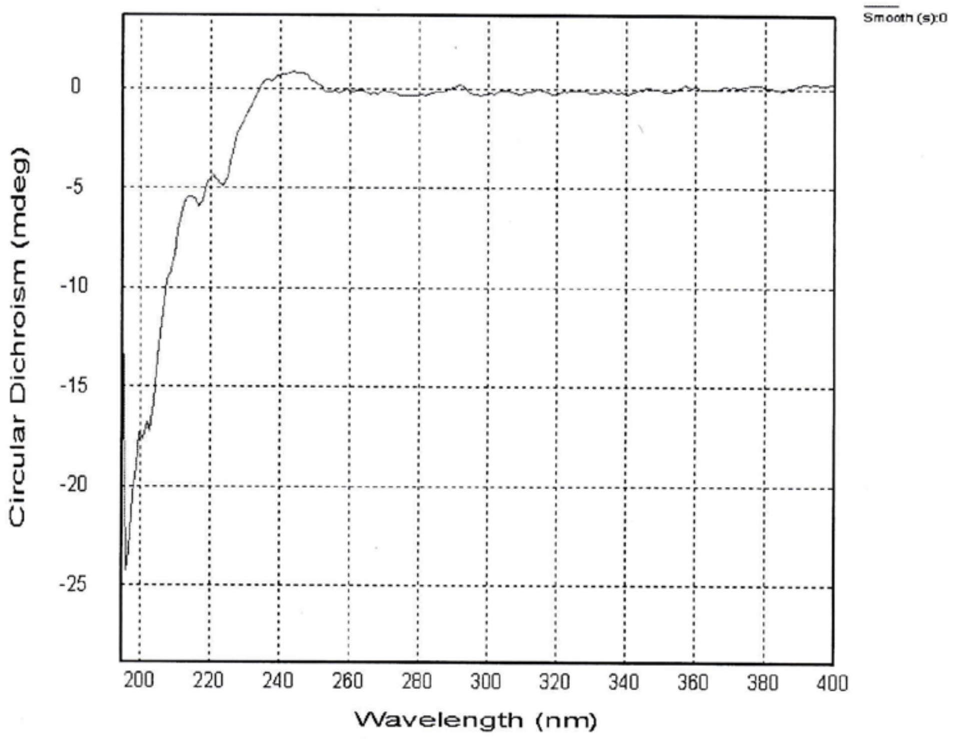

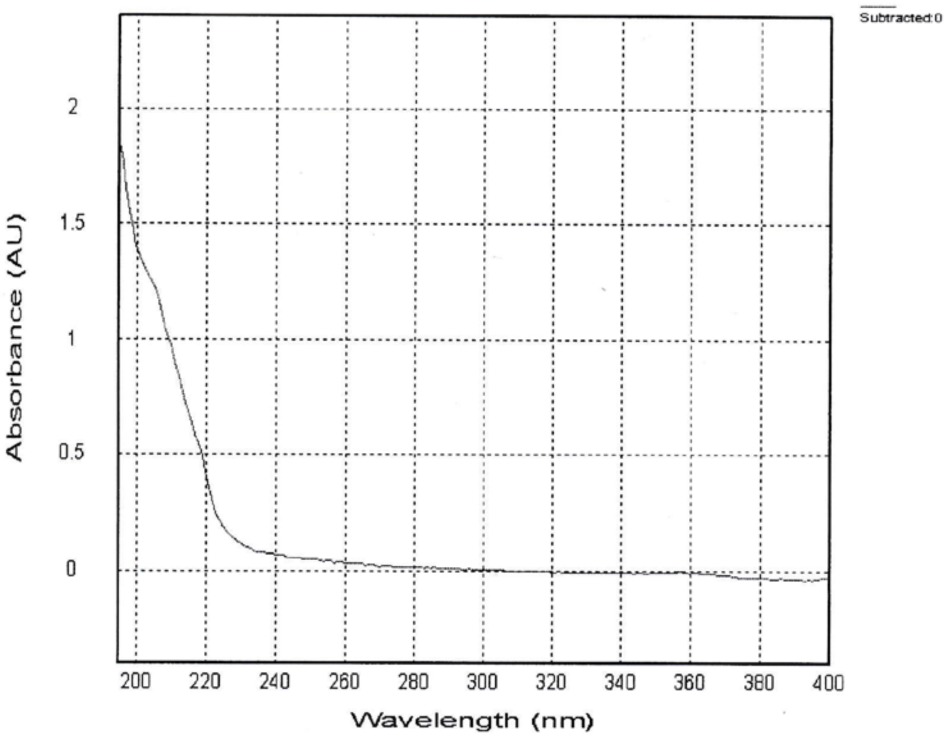

Figure S69. CD spectrum of **7**.
